# Multi-omics characterization of developing forebrain organoids unravels the dynamic molecular events of Rett syndrome pathogenesis

**DOI:** 10.1186/s11689-026-09699-9

**Published:** 2026-04-21

**Authors:** Jarno Koetsier, Nasim Bahram Sangani, Ana Rita Gomes, Maria Margarida Diogo, Tiago G. Fernandes, Freek G. Bouwman, Edwin C. M. Mariman, Mehrnaz Ghazvini, Leon J. Schurgers, Joost Gribnau, Leopold M.G. Curfs, Chris P. Reutelingsperger, Lars M.T. Eijssen

**Affiliations:** 1 Department of Biochemistry, Cardiovascular Research Institute Maastricht (CARIM), Maastricht University, Maastricht, 6200 MD The Netherlands; 2 GKC, Maastricht University Medical Centre, Maastricht, The Netherlands; 3 Department of Bioengineering and iBB-Institute for Bioengineering and Biosciences, Instituto Superior Técnico, Universidade de Lisboa, Lisboa, Portugal; 4 Instituto de Medicina Molecular João Lobo Antunes, Faculdade de Medicina, Universidade de Lisboa, Lisboa, Portugal; 5 Associate Laboratory i4HB – Institute for Health and Bioeconomy, Instituto Superior Técnico, Universidade de Lisboa, Lisbon, Portugal; 6 Department of Human Biology, School of Nutrition and Translational Research in Metabolism (NUTRIM), Maastricht University Medical Centre, Maastricht, The Netherlands; 7 Erasmus MC iPS Facility, Erasmus Medical Center, University Medical Center, Rotterdam, Netherlands; 8 Department of Developmental Biology, Erasmus Medical Center, University Medical Center, Rotterdam, Netherlands; 9 Department of Psychiatry and Neuropsychology, School for Mental Health and Neuroscience (MHeNs), Maastricht University, Maastricht, The Netherlands; 10 Department of Translational Genomics, Maastricht University, Maastricht, The Netherlands

**Keywords:** Rett syndrome, MeCP2, Brain organoids, Transcriptomics, Proteomics, lncRNAs, Spatiotemporal analysis

## Abstract

**Background:**

Rett Syndrome (RTT) is a neurodevelopmental disorder primarily caused by mutations in the *MECP2* gene. Despite its monogenic nature, the molecular events contributing to RTT pathogenesis are not fully elucidated.

**Methods:**

We applied a multi-omics approach to comprehensively analyse the spatiotemporal gene and protein expression patterns in MeCP2-mutant (RTT) and isogenic control (IC) forebrain organoids. Dorsal and ventral forebrain organoids were cultured for 75 days using patient-derived RTT and IC hiPSC lines. Transcriptomics and proteomics profiles were characterized at days 0, 13, 40, and 75, corresponding to distinct neurodevelopmental phases.

**Results:**

The spatiotemporal transcriptomic analysis revealed alterations in GABAergic signaling at the latest neurodevelopmental stages, while changes in neuronal development, DNA-associated processes, and post-transcriptional regulation were found to occur across different stages. These changes were also observed at the protein level and in independent validation datasets. Notably, differentially expressed lncRNA genes such as *MIR137HG* and *PWRN1* may act as regulators of these affected processes. Moreover, our results provide systematic evidence for the involvement of imprinted genes in RTT pathology.

**Conclusions:**

Together, our study lays the foundation for future studies to functionally validate the significance of the identified processes and molecular targets in RTT pathogenesis, and to assess their value as therapeutic targets.

**Graphical Abstract:**

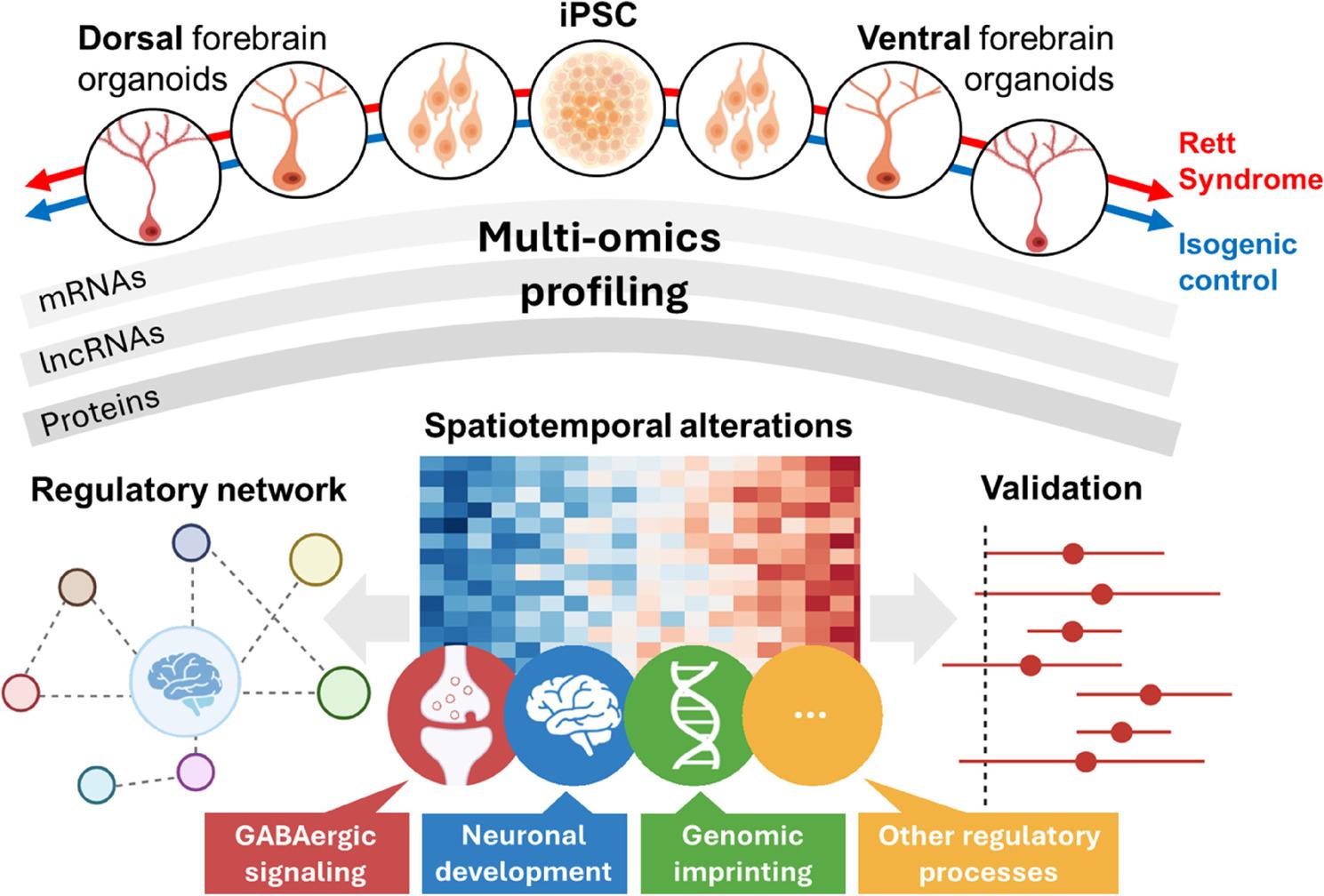

**Supplementary Information:**

The online version contains supplementary material available at 10.1186/s11689-026-09699-9.

## Background

Rett syndrome (RTT, OMIM #312750) - a neurodevelopmental disorder predominantly affecting females - is characterized by a unique pattern of neuroregression following a period of apparently normal development [1]. RTT is primarily caused by mutations in the *methyl-CpG-binding protein 2 (MECP2)* gene, a crucial epigenetic regulator orchestrating gene expression in the central nervous system [2].

To comprehend the complex molecular mechanisms underlying neurological disorders, attention has increasingly turned to high-throughput technologies such as transcriptomic and proteomic analyses. The advantage of these high-throughput technologies lies in their ability to provide a comprehensive snapshot of gene and protein expression patterns, allowing researchers to gain insights into the intricate network of molecular events underlying the pathology. The involvement of MeCP2, which can function both as a transcriptional [3] and post-transcriptional regulator [4, 5], underscores the potential of transcriptomic and proteomic approaches to unravel the regulatory dynamics at play in RTT. For instance, the application of high-throughput approaches in RTT research has, among others, indicated the occurrence of translational dysregulation [5], mitochondrial dysfunction [6, 7] and changes in neural maturation [8] and function [9].

While traditional models such as 2D cell cultures and animal models have been instrumental in advancing our understanding of RTT, their inherent limitations pose challenges in mirroring the complexity of the human brain [10]. The advent of brain organoids − 3D self-organizing structures that recapitulate key features of human brain development – has revolutionized our ability to model neurodevelopmental disorders, providing a platform that more closely resembles the structural and functional intricacies of the human brain compared to conventional models [11]. In contrast to human postmortem brain tissue, which can only be analyzed in a cross-sectional manner, brain organoids derived from iPSCs enable longitudinal investigation of developmental processes within the same experimental system. Although organoids represent a simplified and developmentally immature model that does not fully recapitulate the complexity or progression of the human brain in vivo, they mimic key aspects of early neurodevelopment. This enables investigation of temporal cellular responses to MeCP2 loss-of-function and may help delineate developmental stages that are relevant in RTT pathology. Brain organoids can also be generated in a region-specific manner to identify alterations that are specific to certain brain areas. This is particularly important for RTT, as the expression of MeCP2 and its regulatory targets vary across the different regions of the brain [12, 13]. As exemplified by Gomes et al., RTT pathology indeed manifests differently in ventral and dorsal forebrain organoids [14]. While neurons in the dorsal (glutamatergic) forebrain organoids differentiated prematurely, the neurons in ventral (GABAergic) forebrain organoids exhibited migration defects.

Although RTT has previously been studied in brain organoids [7, 8, 14], no study to date has applied multi-omics techniques to characterize RTT brain organoids in a spatiotemporal manner. This approach could yield in-depth knowledge about how dysfunctions within affected pathways contribute to the onset of RTT. Therefore, our research aims to leverage the power of transcriptomic and proteomic techniques to conduct a comprehensive analysis of spatiotemporal gene and protein expression patterns in MeCP2-mutant (RTT) and matched isogenic control (IC) forebrain organoids. Through this approach, we aim to contribute novel perspectives to the understanding of RTT pathogenesis, potentially paving the way for the development of targeted therapeutic interventions.

## Methods

### hiPSC lines and maintenance

The patient-derived hiPSC cell line (46, XX) with the MeCP2:R255X nonsense mutation (rs61749721) (RTT) and the respective isogenic control (IC) cell line were obtained from Universidade de Lisboa [14]. The ethics approval and written consent for tissue collection were coordinated by Hospital Sant Joan de Déu (HSJD) and Centro Hospitalar de Lisboa Norte (CHLN) Ethics Committees as previously described [14]. Cells were cultured on Matrigel™-coated plates in serum-free mTeSR^TM^1 Plus medium and passaged every 2–3 days using 0.5 mM EDTA dissociation buffer (Thermo Fisher Scientific) when 85% confluency was reached. Furthermore, the iPSC clones were expanded for 2–3 passages and seeded into three technical replicates before beginning the differentiation process. Hence, the three replicates represent separate differentiations to account for the variability in stem cell differentiation.

### Induction of region-specific forebrain organoids

**Fig. 1:**
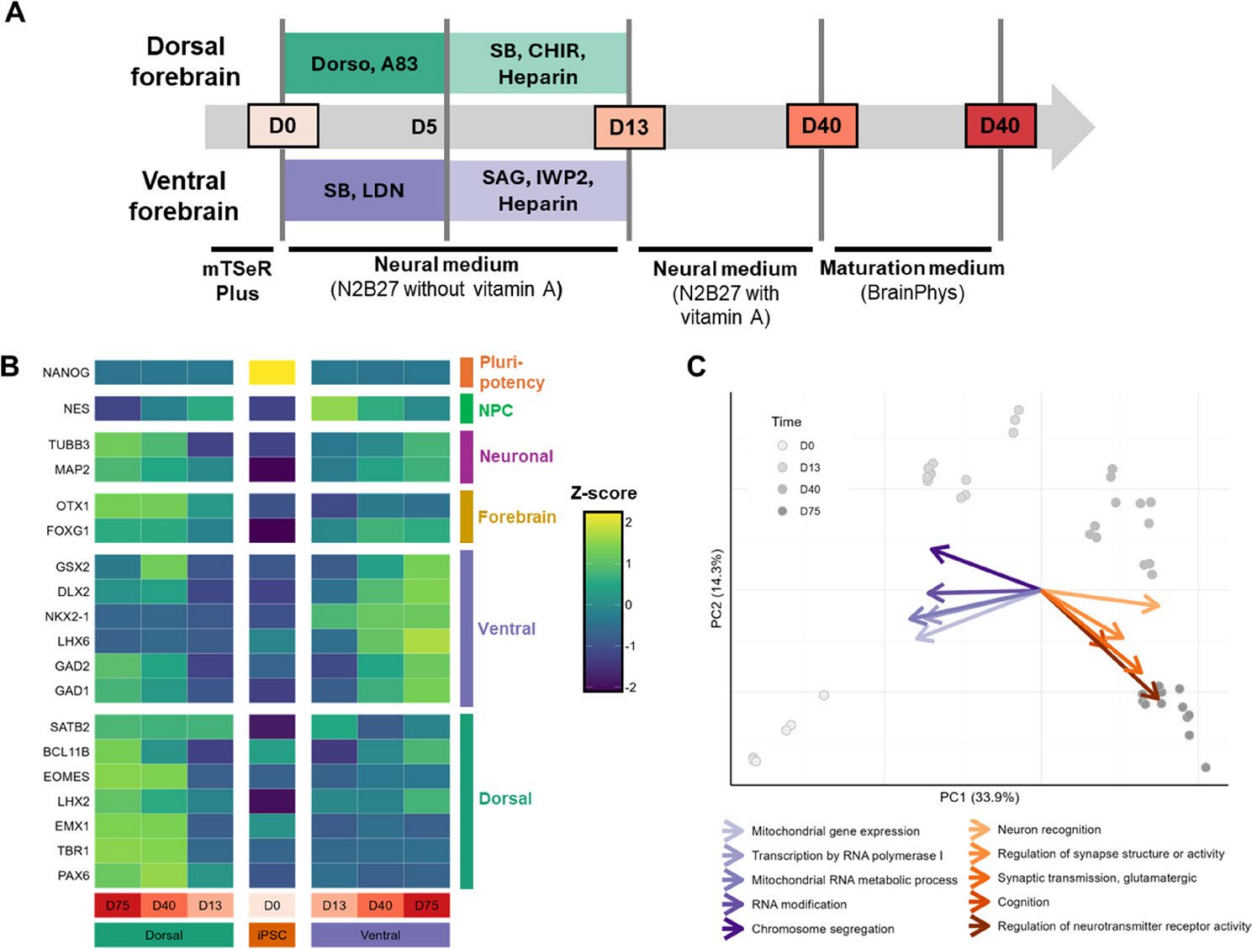
Overview and quality control of the experimental model. **A** Overview of the protocol for the induction, maturation, and maintenance of the region-specific forebrain organoids. RNA and proteins were extracted from dorsal and ventral forebrain organoids at four time points (i.e., day 0, day 13, day 40, and day 75), corresponding to the different developmental stages. **B** Mean expression per time point and region of pluripotency, neural progenitor cell (NPC), neuronal, forebrain, ventral region, and dorsal region markers. The expression values are converted to Z-scores through unit variance scaling. **C** Biplot of the PCA scores (transcriptomics data) and the median loadings of the five gene ontology (GO) terms with highest positive (orange arrows) and negative (purple arrows) normalized enrichment scores (NES). The arrows indicate the GO terms’ contributions to the principal components

To identify the spatiotemporal alterations in the multi-omics profile that occur during neurodevelopment, we applied the protocol for the generation of dorsal (i.e., glutamatergic) and ventral (i.e., GABAergic) forebrain organoids as previously described [14, 15] (Fig. 1A). In brief, hiPSC colonies were incubated with 10 µM ROCK inhibitor (ROCKi, Y-27632, 10 µM, StemCell Technologies) for 30 min at 37 °C, dissociated with Accutase, and seeded in triplicate on microwell plates (AggreWell™ 800, StemCell Technologies) at 1.5 × 10^6^ cells/well in mTeSR^TM^1 Plus supplemented with 10 µM ROCKi for 24 h. The expansion medium was then refreshed entirely without ROCKi supplement. After the aggregates attained a diameter of 250–300 μm (2–3 days), the medium was half-changed to induction medium, marking this as day 0. The neural induction medium (N2B27) comprised of 50% DMEM/F12/N2 (DMEM-F12, (Thermo Fisher Scientific) supplemented with 1% (v/v) N2 (Thermo Fisher Scientific), 1.6 g/L Glucose (Sigma), 1% (v/v) PenStrep, and 20 µg/mL Insulin (Sigma) and 50% of Neurobasal/B27 (i.e., Neurobasal™ medium (Thermo Fisher Scientific) supplemented with 2% (v/v) B27 (without vitamin A)-supplement (Thermo Fisher Scientific), 2 mM L-glutamine (Thermo Fisher Scientific) and 1% (v/v) PenStrep).

For the dorsal aggregates, 2 µM Dorsomorphin (Sigma) and 2 µM A83-01 (Tocris) was added to the medium until day 5, with half-medium being refreshed at days 0, 3, and 5. For the ventral aggregates, the medium was supplemented with 10 µM SB-431,542 (SB) (Sigma) and 100 nM LDN-193,189 (LDN) (Sigma). At day 5, the cell aggregates (approximately 300 aggregates per well of the microwell plate) were transferred to ultra-low attachment 6-well plates (Corning). At day 7, the dorsal forebrain culture medium was half changed with fresh medium supplemented with 1 µM CHIR-99,021 (tebu-bio), 10 µM SB-431,542 (Sigma) and 10 µg/ml Heparin (Sigma) and the ventral forebrain culture medium was half changed with fresh medium supplemented with 2.5 µM IWP2 (Sigma), 100 nM SAG (Millipore) and 10 µg/ml Heparin (Sigma).

### Maintenance and maturation of forebrain organoids

At day 13, the culture medium was half changed with N2B27 (with vitamin A) without adding dorsal (i.e., SB-431542, CHIR-99021, Heparin) and ventral (i.e., SAG, IWP2, Heparin) reagents, continuing until day 40 with the medium being changed every 2–3 days. On day 41, the medium was changed to BrainPhys™ Neuronal Medium (StemCell Technologies), supplemented with NeuroCult™ SM1 Neuronal Supplement (StemCell Technologies), N2 Supplement-A (StemCell Technologies), Recombinant Human Brain Derived Neurotrophic Factor (BDNF, PeproTech, 20 ng/mL), Recombinant Human Glial-Derived Neurotrophic Factor (GDNF, PeproTech, 20 ng/mL), dibutyryl cAMP (1 mM, Sigma), and ascorbic acid (200 nM, Sigma). Organoids were maintained until day 75, changing one third of the total medium every 2–3 days. All organoids in a well (> 20 organoids) were pooled for RNA and protein extraction and quantification.

### RNA extraction and quantification

#### RNA extraction

Samples were collected at day 0 and during differentiation at days 13, 40, and 75. To dissociate the organoids for the purpose of RNA extraction, organoids were first gently dissociated with Accutase (Sigma) for 5 min at 37 °C. Total RNA was extracted using High Pure RNA Isolation Kit (Roche). Samples were then stored at -80 °C until further experiments.

#### Library construction and sequencing

Library construction and sequencing were performed by LC Sciences (Houston, TX, USA). These include the evaluation of RNA integrity by the Agilent Technologies 2100 Bioanalyzer, removal of ribosomal RNA, and RNA fragmentation (divalent cation buffers in elevated temperature). Furthermore, Illumina’s TruSeq-stranded-total-RNA-sample preparation protocol was followed to prepare the sequencing library, while the Agilent Technologies 2100 Bioanalyzer High Sensitivity DNA Chip was used for quality control analysis and quantification of the sequencing library. Finally, paired-ended sequencing was performed with Illumina’s NovaSeq 6000 sequencing system.

#### Preprocessing and quality control

The processing and quality assessment of the sequencing reads was performed by LC Sciences (Houston, TX, USA). Particularly, using *Cutadapt* [16] and in-house scripts, reads with adaptor contamination as well as low quality and undetermined bases were removed. *FastQC* [17] was subsequently utilized to assess the quality of the remaining reads.

As a second step, *Bowtie2* [18] (version 2.5.1) was applied to align the reads to the GRCh38 reference genome. The gene and isoform expression was quantified using *RSEM* [19] (version 1.3.3). Each sample contained 10 to 20 million mapped reads. The established workflow from the *edgeR* R/Bioconductor package [20] (version 3.42.4) was accordingly followed for the filtering of lowly expressed genes and the data normalization. Specifically, genes with an expression above 10 counts per million (CPM) in at least three samples were kept for normalization. Trimmed mean of M values (TMM) and CPM normalization was performed to calculate the normalized expression levels. Boxplots of expression values and Principal Component Analysis (PCA) plots were constructed for the assessment of the pre-processing quality. It should be noted that differential expression analysis results from the X chromosomal genes might be unreliable as the active X chromosome is different between the IC and RTT samples. Hence, X chromosomal genes were excluded from the analysis before normalization.

### Protein extraction and quantification

#### Protein extraction

Proteins were extracted at day 0 and during differentiation at days 13, 40, and 75 in both dorsal and ventral forebrain organoids. To gently dissociate the organoids, they were treated with Accutase (Sigma) for 5 min at 37 °C. After dissociation, protein extraction was performed. The cells were washed twice with cold PBS, collected in a 15 ml tube, and centrifuged at 1500 rpm for 5 min at 4 °C. The resulting pellet was washed with cold PBS, centrifuged again, and excess PBS was carefully removed. The cells were resuspended in 500 µl of 50 mM ammonium bicarbonate (ABC) with 5 M urea. After three freeze/thaw cycles in liquid nitrogen and one minute of vortexing, the suspension was transferred to an Eppendorf tube and centrifuged for 30 min at 14,000 rpm at 10 °C. The resulting supernatant was divided into 200 µl and 100 µl aliquots for protein assays, with the aliquots stored at -80 °C.

Protein concentrations were determined using a Bradford-based protein assay. 50 µg of protein, in 50 µl of 50 mM ABC with 5 M urea were considered. 5 µL of dithiothreitol (DTT) solution (20 mM final) was added and incubated at room temperature for 45 min. The proteins were alkylated by adding 6 µL of iodoacetamide (IAA) solution (40 mM final). The reaction was run at room temperature for 45 min in darkness. The alkylation was stopped by adding 10 µL of DTT solution (to consume any unreacted IAA) and incubated at room temperature for 45 min. For the digestion 2 µg trypsin/lysC was added to the protein and incubated at 37 °C for 2 h. 200 µl of 50 mM ABC was added to dilute the urea concentration and further incubated at 37 °C for 18 h. The digestion mix was centrifuged at 2.500 g for 5 min and the supernatant was collected for liquid chromatography-mass spectrometry (LC-MS).

#### LC-MS

A nanoflow HPLC instrument (Dionex ultimate 3000) was coupled on-line to a Q Exactive (Thermo Fisher Scientific) with a nano-electrospray Flex ion source (Proxeon). Of the digested peptide mixture, 5 µl was loaded onto a C18-reversed phase column (Thermo Fisher Scientific Acclaim PepMap C18 column, 75 μm inner diameter x 50 cm, 2-µm particle size). The peptides were separated with a 240 min linear gradient of 4–45% buffer B (80% acetonitrile and 0.08% formic acid) at a flow rate of 300 nL/min.

MS data were acquired using a data-dependent top10 method, dynamically choosing the most abundant precursor ions from the survey scan (250–1250 m/z) in positive mode. Survey scans were acquired at a resolution of 70,000 and a maximum injection time of 100 ms. Dynamic exclusion duration was 30 s. Isolation of precursors was performed with a 2.0 m/z window and a maximum injection time of 200 ms. The resolution for high energy collision dissociation (HCD) spectra was set to 17,500 and the normalized collision energy was 30 eV. The under-fill ratio was defined as 1.0%. The instrument was run with peptide recognition mode enabled, but exclusion of singly charged and charge states of more than five.

#### Database search and quantification

The MS data were searched using the Proteome Discoverer 2.2 Sequest HT search engine (Thermo Fisher Scientific), against the UniProt human database. The false discovery rate (FDR) was set to 0.01 for proteins and peptides, which had to have a minimum length of 6 amino acids. The precursor mass tolerance was set at 10 ppm and the fragment tolerance at 0.02 Da. One miss-cleavage was tolerated, and the oxidation of methionine was set as a dynamic modification. Carbamidomethylation of cysteines was set as fixed modifications. Label-free quantitation was conducted using the Minora Feature Detector node in the processing step and the Feature Mapper node combined with the Precursor Ions Quantifier node in the consensus step with default settings within Proteome Discoverer 2.2.

#### Data preprocessing and quality control

The pre-processing of the raw proteomics data and the differential expression analysis was done using the *DEP* R/Bioconductor package [21] (version 1.22.0). Similar to the transcriptomics analysis, sex chromosomal proteins were excluded. Furthermore, autosomal proteins were excluded if, in every experimental group (i.e., genotype/region/time combinations), two or three replicates had missing values. Thus, proteins were only included in the analysis if they were quantified in at least two replicates of at least one experimental group. Accordingly, the data was normalized using variance stabilizing normalization (VSN) and mixed imputation was performed for the imputation of missing values. Specifically, k-nearest neighbors (kNN) imputation was done for values missing at random (MAR), while quantile regression imputation of left-censored data (QRILC) was performed for values missing not at random (MNAR). For this, proteins missing in all replicates of at least one experimental group were considered MNAR. Boxplots of the protein expression values and PCA plots were constructed for the assessment of the pre-processing quality. Finally, the proteins (UniProt ID) were matched to their corresponding gene (Ensembl ID and HGNC symbol) using the *biomaRt* R/Bioconductor package [22] (version 2.56.1).

### Evaluation of the spatiotemporal expression profile

To evaluate quality of the induced spatiotemporal differentiation, the mRNA expression of known pluripotency (*NANOG*), neural progenitor cell (*NES*), neuronal (*TUBB3*,* MAP2*), forebrain (*OTX1*, *FOXG1*), ventral region (*GSX2*,* DLX2*,* NKX2-1*,* LHX6*,* GAD1*,* GAD2*), and dorsal region (*SATB2*,* EOMES*,* BCL11B*,* LHX2*,* EMX1*,* TBR1*, *PAX6*) markers was evaluated [14]. The expression for each gene was converted to a z-score through unit variance scaling. Furthermore, organoid sections (day 41) were prepared and immunostained as previously described [14, 15]. The dorsal and ventral organoid sections were immunostained for TBR1 (Rabbit anti-TBR1; Millipore) and FOXG1 (Rabbit anti-FOXG1; Cell Signaling Technology), respectively. Goat anti-Rabbit IgG, Alexa Fluor^TM^–546 (Thermo Fisher Scientific) was used as the secondary antibody. DAPI (Sigma) was used for nuclear counterstaining.

To further evaluate the spatiotemporal expression profile in the transcriptomics dataset, analysis of the PCA loadings was performed to characterize the genes with a time-dependent expression profile. As PC1 captured most of the variation over time, the loadings of PC1 were used to rank the genes for the Gene Ontology (GO) gene set enrichment analysis (GSEA). In short, GSEA evaluates whether the genes of a GO term are non-randomly distributed in the ranked gene list. Particularly, a negative enrichment score indicates that the genes of the corresponding GO term are more prevalent among the genes with a negative PC1 loading, while a positive enrichment score denotes a higher abundance of positively loaded genes, both pointing at time-dependent expression profiles for these terms. The GSEA was performed using the *clusterProfiler* R/Bioconductor package [23] (version 4.8.3). To avoid redundancy of GO terms, we only focused on the Biological Process (GO-BP) aspect, and the *rrvgo* R/Bioconductor package [24] (version 1.12.2) was applied to cluster similar GO terms using Resnik similarity (threshold = 0.85) with the most significant GO term as the representative term for the cluster. Similar to the transcriptomics data, PC1 of the proteomics data captured most of the time-dependent variation and the proteins with the largest negative and positive PC1 loadings were considered to have a time-dependent expression profile.

Due to the absence of vasculature in the forebrain organoid model, apoptosis may occur at later developmental stages. Hence, the protein expression levels of known pro-apoptotic markers BAX, CASP3, and CASP6 and anti-apoptotic marker BAG1 were evaluated to assess the potential presence of an apoptotic expression profile across the neurodevelopmental stages.

### Identification of RTT-specific alterations

#### Identification of protein markers

To identify proteins with an altered expression profile in RTT at each time point and brain region, differential expression analysis was performed using the *DEP* R/Bioconductor package [21] (version 1.22.0), which applies the *limma* R/Bioconductor package [25] (version 3.56.2) to fit protein-wise linear regression models. Proteins with an FDR-adjusted P value < 0.05 and absolute log_2_ fold change (|log_2_FC|) > 1 were considered differentially expressed. Accordingly, we identified proteins with particular time- and region-specific alterations by applying selection criteria as described in Table S1, Additional file 1.

In a separate analysis, we aimed at identifying proteins with a transient, early time differential expression profile. These transient proteins markers were defined as being differentially expressed at day 13 (FDR-adjusted P value < 0.05 and |log2FC| > 1), while remaining unchanged at days 40 and 75 (nominal P value > 0.1). To compare protein and gene expression profiles, proteins and their corresponding genes were matched using *biomaRt* R/Bioconductor package [22] (version 2.56.1).

#### Trajectory inference analysis

Trajectory inference was performed using genes annotated to the forebrain development GO term (GO:0030900). PCA was conducted on IC samples only to define a reference developmental trajectory. A smoothing spline was fitted through the IC samples in PCA space to model the progression of forebrain development. RTT samples were subsequently projected into the same PCA space, and pseudotime values were assigned by identifying the closest point on the reference spline. Pseudotime was defined as the distance along the spline. Trajectory inference was conducted separately for dorsal and ventral forebrain organoids. Differences in pseudotime between RTT and IC samples at each time point were assessed using Welch’s two-sample t-test.

#### Identification of alterations in GABAergic and glutamatergic signaling 

To investigate how GABAergic signaling in the ventral (i.e., GABAergic) region and glutamatergic signaling in the dorsal (i.e., glutamatergic) region were affected in RTT, we visualized the signed -log_10_ P value (Eq. 1) of GABAergic and glutamatergic signaling-associated genes over time in the ventral and dorsal region, respectively. Particularly, for this analysis, genes involved in GABA or glutamate synthesis, transport, and reception were selected from the *GABA receptor signaling (WP4159)* and *Neuroinflammation and glutamatergic signaling (WP5083)* pathways from WikiPathways [26], respectively.

#### Identification of altered biological processes

For the transcriptomics data, instead of identifying individual markers, we focused on characterizing the altered biological processes in the RTT organoids. For this, the quasi-likelihood F-test from the *edgeR* R/Bioconductor package [20] (version 3.42.4) was first applied to assess the statistical significance of the gene expression difference between the RTT and IC at each time point (i.e., days 0, 13, 40, and 75) per region (i.e., ventral and dorsal region). Subsequently, GO-BP GSEA (*clusterProfiler* R/Bioconductor package [23], version 4.8.3) was performed at each time point and brain region separately using the signed -log_10_ P value to rank the genes (Eq. 1).

Equation 1 $$\:Ranking\:Score=\:-{log}_{10}\left(P\:value\right)\cdot\:sgn\left({log}_{2}FC\right)$$

To avoid redundancy of GO terms, the *rrvgo* R/Bioconductor package [24] (version 1.12.2) was applied to cluster similar GO terms using Resnik similarity (threshold = 0.8). To be able to jointly represent the results from the different time points and brain regions, the GO term with the highest mean -log_10_ P value over all time points and brain regions was chosen as the representative term.

Despite the lower coverage of the proteomics compared to the transcriptomics data, it is still purposeful to see whether the altered biological processes at the transcriptomics level are also reflected at the protein level. Hence, we also performed GSEA on the proteomics data using signed -log_10_ P value as the ranking variable (Eq. 1) for the significant GO-BP terms identified from the transcriptomics data.

### Validation and interpretation of the alterations in RTT

#### Validation in publicly available RNA-seq data

To further validate our GO-BP GSEA results, we searched the Gene Expression Omnibus (GEO) [27] website (keyword: “Rett Syndrome”) for RNA sequencing data of RTT and control samples (species: homo sapiens; minimal sample size per experimental group: 3) (Table 1). The raw count tables of four studies (i.e., GSE107399 [28] GSE123753 [5] GSE117511 [9] and GSE128380 [29]) were downloaded from GEO. For each validation dataset separately, the lowly expressed genes were filtered out and the raw counts were normalized using the *edgeR* R/Bioconductor package [20] (version 3.42.4). We applied the same filtering and normalization settings as for our own dataset. This package was also used to perform differential expression analysis. Depending on the study design, a different statistical model was fitted to assess the statistical significance of the expression difference between RTT and control samples (Table 1). GO-BP GSEA was performed for each dataset using the signed -log_10_ P value to rank the genes (Eq. 1). However, for the validation, we only focused on the significant GO-BP terms identified from our transcriptomics data.

**Table 1:** Overview of the RNA-seq validation sets

GEO accession (PubMed ID)	Origin/tissue	MeCP2 mutation (domain*)	Design	Contrasts**
GSE107399 (PMID: 29742391)	Cell culture: induced pluripotent stem cells (iPSCs), neural progenitor cells (NPCs), and neurons.	X487W (CTD) or E235fs (TRD)	Expr ~ 0 + (genotype * cell type) + patient	RTT.iPSC – WT.iPSC RTT.NPC – WT.NPC RTT.Neuron – WT.Neuron
GSE123753 (PMID: 32209477)	Cell culture: NPCs and neurons.	Deletion of exons 3 and 4 (NTD, MBD, ID, TRD, and CTD)	Expr ~ 0 + (genotype * cell type)	RTT.NPC – WT.NPC RTT.Neuron – WT.Neuron
GSE117511 (PMID: 32526163)	Interneurons at different days of maturation: D0, D10, D18, D32, D46, and D74.	R133C (MBD)	Expr ~ 0 + genotype + time	RTT – WT
GSE128380 (PMID: 32561870)	Post-mortem brain: temporal cortex (TC) and cingulate cortex (CC).	T158M (MBD), exon deletion, or non-reported mutation.	Expr ~ 0 + (genotype * brain region)	RTT.TC – WT.TC RTT.CC – WT.CC

*NTD N-terminal domain, *MBD* methyl binding domain, *ID* inter domain, *TRD* transcriptional repression domain, *CTD* C-terminal domain. **RTT Rett syndrome/MECP2-mutant, *WT* wild type/control

#### Validation in publicly available proteomics data

To validate the findings from our proteomics data, the proteomics dataset of Varderidou-Minasian et al. (PXD013327) [30] was re-analyzed. This validation set includes the protein quantification of RTT and IC iPSC-derived NPCs at four time points during neuronal differentiation (days 3, 9, 15, and 22). Data pre-processing and differential expression analysis was performed using the *DEP* R/Bioconductor package [21] (version 1.22.0) as described for our proteomics data. The signed -log_10_ P value was used as the ranking variable for the GSEA (Eq. 1).

#### Permutation analysis

To evaluate whether the number of GO-BP terms that reached statistical significance in the proteomics data and transcriptomics validation sets is more than expected by chance, a 1,000-permutation analysis was performed where, for each permutation, the genes/proteins were randomly ordered before performing GSEA. Particularly, for each permutation of the transcriptomics data, a randomly ordered gene list was generated by randomly selecting a list of genes from the union of genes that pass quality control in at least one validation set. The length of the selected gene list was equal to the mean number of genes that passed QC in the different datasets. For the permutation analysis of the proteomics data, all proteins that passed QC were randomly ordered at each permutation. The number of significant GO terms for each validation set was compared against the null distribution generated by the permutation analysis.

#### Validation in publicly available ChIP-seq data

In order to assess whether the identified GO-BP terms are transcriptionally regulated by MeCP2, we used the publicly available mouse ChIP-Seq dataset (GSE71126 [31]) to quantify MeCP2 binding to the promoter region (+/- 3000 base pairs from the transcription start site (TSS)) of the genes annotated to the significant GO-BP terms identified from our transcriptomics data. The MeCP2 enrichment was quantified around the gene’s TSS (i.e., TSS +/- 3000 bp). The Welch two-sample t-test was applied to compare the mean level of MeCP2 enrichment of the GO-BP term’s genes versus all other genes.

#### Interpretation of the altered biological processes

To understand the spatiotemporal expression profile of significant GO-BP terms, the eigengene and eigenprotein of the GO terms were calculated and visualized. The eigengene was defined as the first principal component of the gene expression data of the genes in the corresponding GO term. Similarly, the eigenprotein was defined as PC1 of the corresponding GO term’s protein expression data. The extent to which the individual genes/proteins in the term contribute to its eigengene’s/eigenprotein’s spatiotemporal expression profile was defined by their module membership. The module membership was calculated as the absolute Pearson correlation coefficient of the gene/protein expression values with the eigengene/eigenprotein.

#### Differential isoform usage analysis 

To assess changes in isoform usage, differential isoform usage (DIU) analysis was performed. We only included isoforms with a total raw gene count above 50 in all samples and a raw isoform count above 10 in all RTT or in all IC samples. The relative abundance of each isoform (i.e., ratio of isoform expression and total gene expression weighted by the transcript’s effective length) as estimated by RSEM [19] (version 1.3.3) was compared between RTT and IC using the Mann-Whitney U Test. A gene was considered to undergo DIU in case of a Mann-Whitney U test P value ≤ 0.1 at minimally four of the seven time points and/or brain regions. For the Mann-Whitney U test, a P value threshold of 0.1 was chosen because *P* ≤ 0.05 cannot be reached for a sample size of three per experimental group.

#### Differentially regulated post-transcriptional events

To identify differentially regulated post-transcriptional events, a linear model was fitted with the protein expression (log_2_ intensity) as the dependent variable and the gene expression (log_2_ CPM) and experimental group (RTT and IC) as the independent variables. Genes were considered to be subject to differentially regulated post-transcriptional events when the coefficient for the gene expression is significantly higher than 0 and the coefficient for the experimental group is significantly different from 0 (FDR-adjusted P value < 0.05). These criteria imply that we looked for genes whose protein expression depends on their gene expression, and that the intercept of this relationship is significantly different for the two groups.

### Identification of protein and lncRNA regulators

To identify lncRNA and protein regulators of the significant GO-BP terms from the transcriptomics analysis, we applied a tree-based network inference method (*GENIE3* R/Bioconductor package [32], version 1.22.0) with the eigengenes of the GO-BP terms as the target variables. When performing network inference, we limited potential regulators to proteins and lncRNAs. Specifically, we only used proteins that have been reported to act as a transcription factor [33] and lncRNAs that are known to physically interact with the DNA or a transcription factor protein (NPInter [34], version 5.0). The top 5% of the protein-eigengene interactions and the top five permille of the lncRNA-eigengene interactions were used to construct the regulatory network.

### Imprinted genes

#### Evaluation of the differential expression profile of imprinted genes

Since loss-of-imprinting has been suggested to be involved in RTT pathology [35], we characterized the differential expression profile of previously reported or predicted imprinted genes (*geneimprint.com*). Specifically, the Fisher’s Exact test was applied to assess whether the odds of a gene being differentially expressed (i.e., FDR-adjusted P value < 0.05 and |log_2_FC| > 1) was significantly different for imprinted genes than for non-imprinted genes. The Fisher’s Exact test was chosen to accommodate for the low frequency of imprinted genes.

#### Validation of the differential expression profile

The differential expression profile of imprinted genes was validated in the publicly available RNA-seq datasets (Table 1). However, as the number of differentially expressed genes in these validation datasets was limited, we used the nominal P value instead of the FDR-adjusted P value to establish the differential expression status of a gene. Individual effects of the different validation sets were pooled by fitting a random effects model (inverse variance method, restricted maximum-likelihood estimator, Hartung-Knapp adjustment) using the *meta* package [36] (version 7.0.0).

#### Evaluation of MeCP2 binding to the promoter of imprinted genes

We used the publicly available mouse ChIP-Seq dataset (GSE71126 [31]) to quantify MeCP2 binding to the promoter region (+/- 3000 base pairs from the TSS) of imprinted and non-imprinted genes. The imprinted genes for the mouse species were retrieved from *geneimprint.com*. The Welch two-sample t-test was applied to compare the mean level of MeCP2 enrichment between imprinted and non-imprinted genes.

#### Allele-specific expression analysis

Using our RNA-seq data, allele-specific expression (ASE) analysis was performed to investigate the loss or gain of bi-allelic expression of imprinted genes based on heterozygous genotype calls in these genes. ASE was performed using the *CollectAllelicCounts* function of the Genome Analysis Toolkit (GATK, version 4.5.0) [37]. The ASE score for a specific single nucleotide polymorphisms (SNP) was defined as the ratio between read count of the highest expressed allele and the total read count, as described before [38]. Here, an ASE score of 0.5 corresponds to a bi-allelic expression with 50% of the reads coming from either allele, while a score of 1 indicates a purely mono-allelic expression. To limit the potential bias introduced by spontaneous mutations during the generation and maintenance of the brain organoids, we limited our ASE analysis to the SNPs from the Axiom Exome Plus genotypes VCF file (genomics-public-data, GATK resource bundle [37]) which include coding variants observed multiple times in different sequencing datasets.

A gene was considered bi-allelically expressed if it has an ASE score < 0.85 with reference and alternative allele count > 5 in all samples of at least four time points and/or brain regions. Genes with a bi-allelic expression in either the RTT or IC group were considered to have a differential ASE in the case of Mann-Whitney U Test P value ≤ 0.1 at minimally four time points and/or brain regions.

The ASE results were validated in mouse RNA-Seq data (GSE71126 [31]). The ASE validation analysis was limited to the mouse genome collection of SNPs (REL2021) from the Mouse Genomes Project [39]. For this validation, a gene was considered bi-allelically expressed if it has an ASE score < 0.85 with reference and alternative allele count > 5 in all samples of an experimental group (i.e., RTT and wild-type (WT)). Genes with a bi-allelic expression in either the RTT or WT group were considered to have a differential ASE in case of Mann-Whitney U Test P value ≤ 0.1.

## Results

### The multi-omic profile captures neurodevelopmental processes with a dynamic spatiotemporal expression

In order to study the spatiotemporal expression profile of the developing brain, both dorsal and ventral forebrain organoids were cultured for 75 days using a patient-derived hiPSC cell line (46, XX) with the MeCP2:R255X nonsense mutation (RTT) and the respective isogenic control (IC) cell line. Transcriptomic and proteomic profiles were characterized at days 0, 13, 40, and 75 (Fig. 1A). The quality control of the transcriptomics and proteomics data demonstrated high data quality (Figure S1, Additional file 1), the absence of an enhanced pro-apoptotic protein expression profile at the latest developmental time points (Figure S2A, Additional file 1), and mono-allelic expression of the *MECP2* gene (Figure S3, Additional file 1). Furthermore, the region- and/or time point-specific markers [14] exhibited the desired spatiotemporal expression patterns (Fig. 1B), which was further confirmed by immunofluorescence imaging (Figure S4, Additional file 1). Gomes et al. demonstrated that RTT (MeCP2:R255X) and IC ventral and dorsal forebrain organoids are electrophysiologically active and contain astrocytes [14]. The latter was supported by our transcriptomics data, which showed elevated expression levels of the astrocyte markers *GFAP*,* S100B*, and *SOX9* at later neurodevelopmental stages (Figure S5, Additional file 1).

For both the transcriptomics and proteomics data, the first principal component (PC1), which captures the largest amount of variance in the data, distinguished the different neurodevelopmental stages, with higher PC1 scores indicating more advanced developmental time points (Fig. 1C and Figure S2B, Additional file 1). Therefore, to identify the biological processes that exhibit time-dependent transcriptional changes, gene set enrichment analysis (GSEA) was conducted on the PC1 loadings of the transcriptomics data. This analysis showed that DNA- and RNA-associated processes, such as *Transcription by RNA polymerase I*, *RNA modification*, and *Chromosome segregation* are particularly active during the early time points (Fig. 1C). In contrast, neuronal differentiation-associated processes, including *Neuron recognition*, *Regulation of synaptic structure or activity*, and *Cognition* have a high gene expression at the later neurodevelopmental stages (Fig. 1C). The temporal expression profile of these processes are confirmed by the expression data of the LIBD Stem Cell Browser [40] (Figure S6, Additional file 1). Similarly, in the proteomics data, the proteins with an increased expression at the latest neurodevelopmental stages include proteins (i.e., MAP1B, DPYSL2, NCAM1) that are enriched in the brain (proteinatlas.org [41], version 23.0), while those with a lower expression at these later stages solely encompassed ribosomal proteins (i.e., RPL5, RPL3, RPS3A, RPS26, RSL1D1) that do not exhibit a tissue-specific expression profile (Figure S2B, Additional file 1).

### RTT organoids exhibit region- and time-specific gene and protein expression alterations

**Fig. 2:**
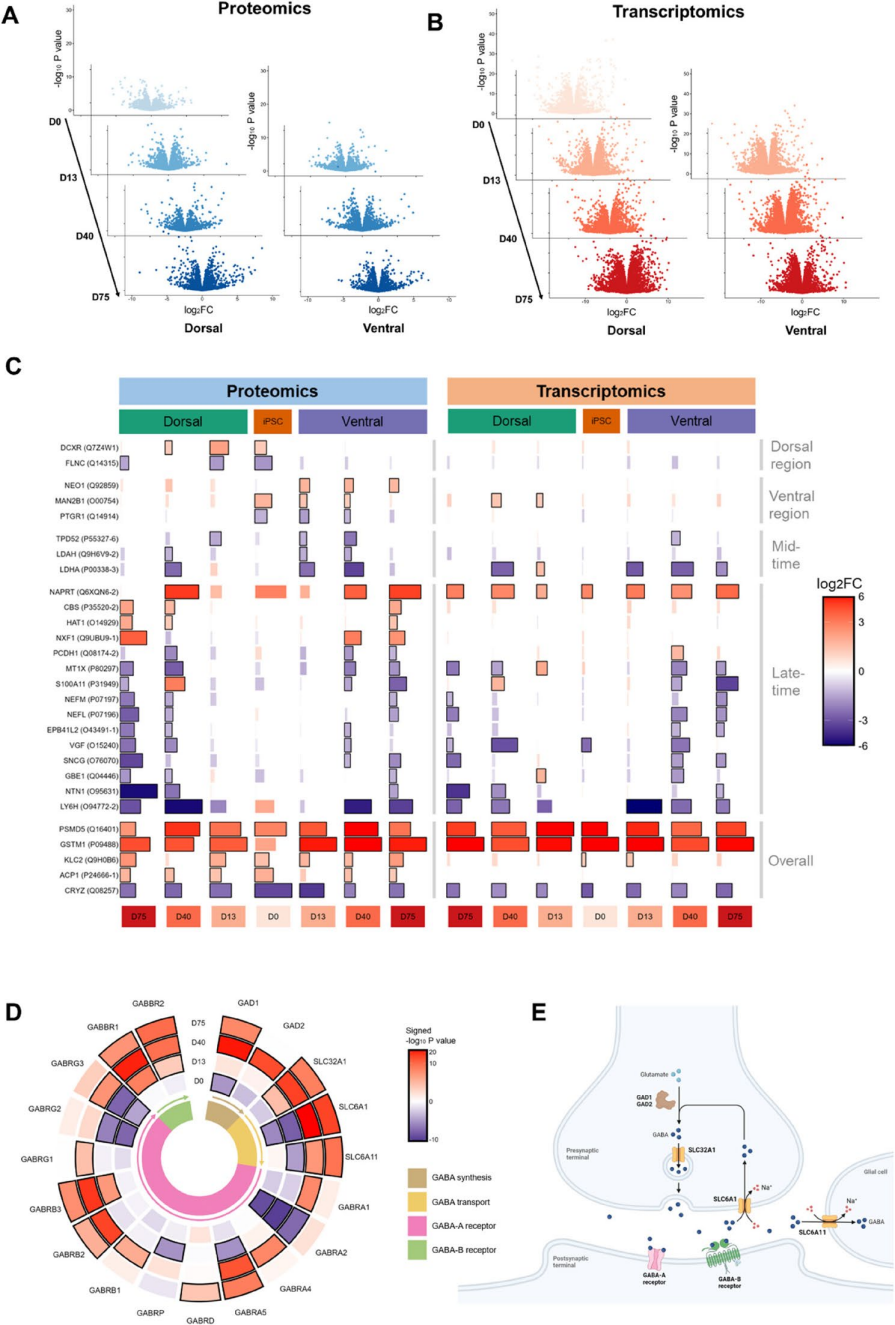
Characterization of the spatiotemporal alterations in RTT.** A** Volcano plots of the proteomics data for the comparison RTT vs. IC at each time point and brain region. **B** Volcano plots of the transcriptomics data for the comparison RTT vs. IC at each time point and brain region. Note that, in the volcano plots (panel A and B), the D0/iPSC comparison is the same for the ventral and the dorsal region. **C** Bar chart of the log_2_FCs of the overall and region/time-specific protein markers at the different time points and/or brain regions (left). The log_2_FCs of the corresponding genes are shown in the right panel. The bar size corresponds to the absolute log_2_FC, and the black border around the bars indicates an FDR-adjusted P value < 0.05 and |log_2_FC| > 1. **D** Circular heatmap of gene expression changes in GABAergic signaling-associated genes colored by the signed -log_10_ P value (i.e., -log_10_ P value * sign log_2_FC) for the comparison RTT vs. IC. A consistent gene expression upregulation in RTT is seen at days 40 and 75. The black border around the heatmap tiles indicates an FDR-adjusted P value < 0.05. **E** Schematic overview of the GABAergic signaling pathway and their associated genes. The pathway was created with BioRender.com

Depending on the brain region and time point, the number of differentially expressed genes (i.e., FDR-adjusted P value < 0.05 and |log_2_FC| > 1) for the “RTT versus IC” statistical comparison varied between 1,735 and 3,649, while the number of differentially expressed proteins (i.e., FDR-adjusted P value < 0.05 and |log_2_FC| > 1) ranged between 67 and 304 (Fig. 2A-B and Figure S7, Additional file 1). From the differentially expressed proteins, we identified five species that were differentially expressed with a consistent direction of effect at minimally six of the seven time points and/or brain regions (i.e., PSMD5, GSTM1, KLC2, ACP1, and CRYZ).

Although there were no proteins with an early-time differential expression profile (i.e., differentially expressed at day 0 and day 13 in both regions), we identified 18 neural progenitor cell (NPC)-specific protein markers that are specifically differentially expressed at day 13, but not at the later developmental stages (Figure S8, Additional file 1). Among these 18 transiently differentially expressed proteins are multiple neurodevelopmental (e.g., ROBO2, SS18L1, PPP2R5D, and CYFIP2) and mitochondrial (e.g., NDUFS7, MICOS13, and NDUFA6) proteins. Furthermore, we identified several proteins with mid- and late-time changes as well as proteins with region-specific (ventral or dorsal) alterations (Fig. 2C). The protein expression profiles of 3,341 of the 3,428 proteins that passed QC were successfully mapped to their corresponding gene expression profiles. The median Spearman correlation between gene and protein expression of gene-protein pairs was 0.51, with 16% of the pairs exhibiting a negative correlation (Figure S9, Additional file 1). Notably, many of the identified protein markers in Fig. 2C had a similar differential expression profile at the transcriptomics level.

### GABAergic expression profile is altered in the RTT ventral region

Since synaptic dysfunction is known to be central in RTT pathology [1], we also investigated the transcriptomic changes in GABAergic signaling in the ventral (i.e., GABAergic) region and glutamatergic signaling in the dorsal (i.e., glutamatergic) region. At the late time points (i.e., days 40 and 75), when neurons appear the ventral region forebrain organoids, there was an increased expression in RTT of the genes involved in GABAergic signaling (Fig. 2D-E). In line with these findings, *DLX5-AS*, a positive regulator of GABAergic signaling [42], was found to be upregulated, while the negative regulator *BDNF* [43] had a downregulated mRNA expression in the ventral region (Figure S10, Additional file 1). In contrast, no consistent up- or downregulation of glutamatergic signaling-associated genes was identified in the dorsal region (Figure S11, Additional file 1).

### RTT organoids show transcriptomic and proteomic changes in chromatin remodeling, post-transcriptional regulation, and neuronal development

**Fig. 3:**
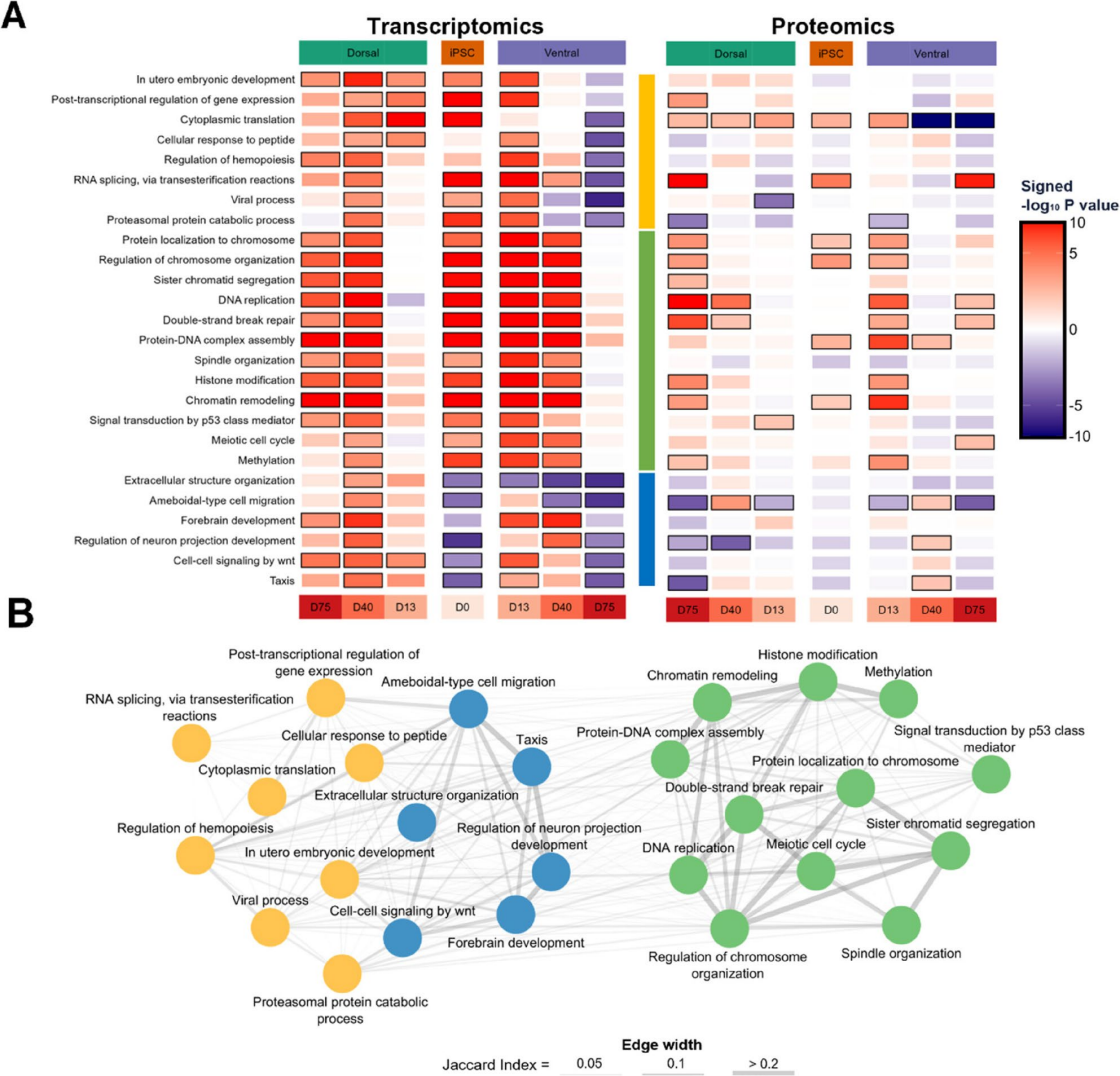
Gene Ontology (GO) Gene Set Enrichment Analysis (GSEA) results of RTT versus IC forebrain organoids. **A** The heatmap of the transcriptomics results (left) shows the signed -log_10_ P value of the GO terms with an FDR-adjusted P value < 0.05 at minimally four time points and/or brain regions. The GO terms fall into three broad categories: (1) neuronal migration and development-related processes (blue cluster), (2) DNA-associated processes (green cluster), and (3) post-transcriptional and other processes (yellow cluster). The sign of the signed -log_10_ P value is determined by the enrichment score, where a negative and a positive score indicate an overrepresentation of the GO term’s genes among the down- and upregulated genes, respectively. The black border around the heatmap tiles indicates an FDR-adjusted P value < 0.05. The GSEA heatmap on the right shows the signed -log_10_ P value of the GO terms from our proteomics analysis. In this heatmap, a P value < 0.05 is indicated by a black border around the heatmap tiles. **B** Network diagram showing the overlap between GO terms, quantified using the Jaccard index. The Jaccard index is the number of shared genes between two GO terms divided by the total number of unique genes present in either term (i.e., the set size of the intersection divided by the set size of the union). The edge width is proportional to the Jaccard index, with thicker edges indicating greater gene overlap. The node colors correspond to the three GO categories from panel A

After the differential expression analysis, GSEA was performed to identify the biological processes with altered gene expression levels between RTT and IC organoids. This analysis led to the identification of 26 Gene Ontology – Biological Process (GO-BP) terms with an FDR-adjusted P value < 0.05 at minimally four of the seven time points and/or brain regions. As shown in Fig. 3, these terms fall within three broad categories: (1) neuronal migration and development-related processes (blue cluster), (2) DNA-associated processes (green cluster), and (3) post-transcriptional and other processes (yellow cluster). In addition to these GO-BP terms with an overall differential expression profile, we identified several GO-BP terms that are specific to the late time points (e.g.,* Positive regulation of cytokine production*), mid time points (e.g.,* Response to radiation*), ventral region (e.g.,* Cell-substrate adhesion*), and dorsal region (e.g.,* Purine-containing compound metabolic process*) (Figures S12 and S13, Additional file 1). Despite the lower coverage, at most time points and/or brain regions, minimally six of the 26 GO-BP terms with an overall differential expression profile reached statistical significance (P value < 0.05) in the proteomics data (Fig. 3). Using a 1000-permutation analysis, we estimated that the probability of having at least six significant GO-BP terms for randomly labeled proteomics data is 9e-3. Notably, some of the changes might be caused by a different developmental timing of RTT and IC forebrain organoids (Figure S14, Additional file 1).

### Independent data validate the identified transcriptomic and proteomic changes

**Fig. 4:**
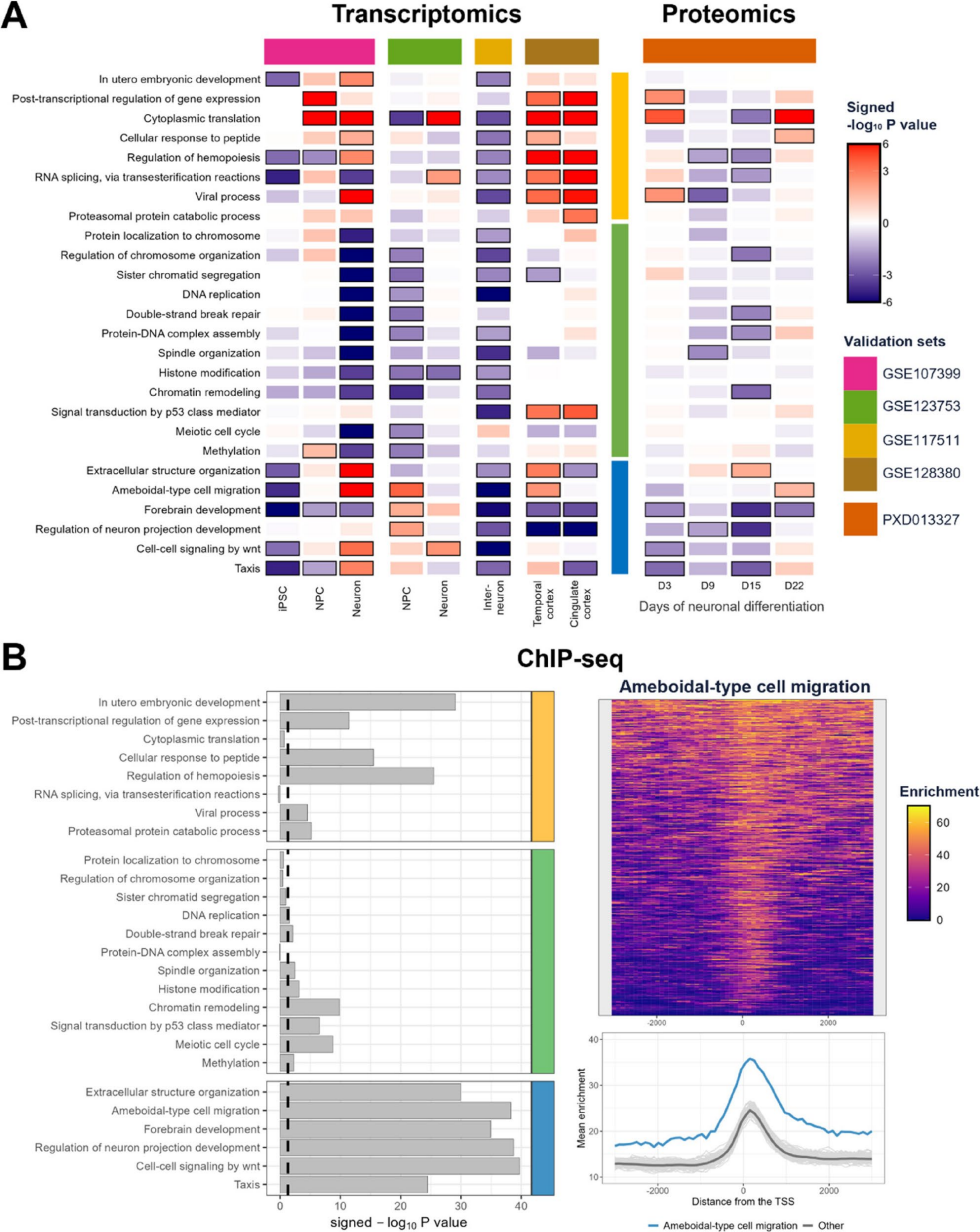
Validation of the GSEA results. **A** The heatmap shows the signed -log_10_ P value of GSEA in the transcriptomics validation sets (left panel) and proteomics validation set (right panel) for the 26 GO-BP terms shown in Fig. 3. The GO terms fall into three broad categories: (1) neuronal migration and development-related processes (blue cluster), (2) DNA-associated processes (green cluster), and (3) post-transcriptional and other processes (yellow cluster). The black border around the heatmap tiles indicates a P value < 0.05. **B** The left panel shows the statistical significance (i.e., -log_10_ Welch two sample t-test P value) of the mean MeCP2 enrichment around the TSS (+/- 3000 bp) of the genes annotated to the corresponding GO term versus all other genes. The dashed line indicates the P value threshold of 0.05. The top right panel shows the MeCP2 enrichment of the genes annotated to the *Ameboidal-type cell migration (GO:0001667)* term. The mean MeCP2 enrichment of these genes (blue) versus the enrichment of 100 random gene sets (grey) is visualized in the bottom right panel

To validate our GO-BP GSEA results, we characterized RTT-associated alterations in independent RTT RNA-seq datasets from male (GSE117511 [9]) and female (GSE107399 [28] and GSE123753 [5]) neural cell cultures and postmortem brains (GSE128380 [29]) as well as in a RTT proteomics dataset from iPSCs undergoing neuronal differentiation (PXD013327 [30]). Interestingly, the identified 26 GO-BP terms with significant changes at minimally four of the seven time points and/or brain regions (Fig. 3) also showed significant alterations in the different transcriptomics and proteomics validation sets (Fig. 4A). Specifically, permutation analysis revealed that the probability of having at least six of the 26 significantly enriched GO-BP terms is approximately 3e-3 for randomly labeled transcriptomics data and 4e-3 for randomly labeled proteomics data, indicating that the observed findings are unlikely to have occurred by chance. However, the direction of effect is not always consistent between the different transcriptomics and proteomics validation sets.

Among the identified GO terms, *Cytoplasmic translation (GO:0002181)* reached statistical significance in most of the transcriptomics and proteomics datasets (Figs. 3 and 4A). Specifically, the protein and RNA expression of cytoplasmic translation-related genes was slightly increased in the dorsal/glutamatergic region, whereas its expression was more pronouncedly decreased at the latest time points of the ventral/GABAergic region. Interestingly, the changes in this GO term are particularly driven by the expression alterations of the small and large ribosomal subunit proteins (Figures S15 and S16, Additional file 1).

Given the changes in the gene and protein expression of *RNA splicing*,* via transesterification reactions (GO:0000375)*, we sought to investigate whether we could observe differential isoform usage (DIU) in the RTT forebrain organoids. Through the DIU analysis, we identified 443 genes with changes in the relative isoform expression level at minimally four time points and/or brain regions. Interestingly, 216 of these 443 DIU genes were also differentially expressed (i.e., FDR-adjusted P value < 0.05) at minimally four time points and/or brain regions. Additionally, 15 of the 26 GO terms of Fig. 3 were significantly enriched with the DIU genes, with *Cytoplasmic translation (GO:0002181)* being the most significant (Figure S17, Additional file 1). Together, these findings indicate that alterations in the relative isoform distribution in RTT are often associated with gene expression changes, where not all of the gene’s isoforms contribute equally to the increasing or decreasing expression levels. This can, among others, be seen for the ribosomal protein gene *RPL9*, where predominantly the protein-coding RPL9-201 and − 203 isoforms are responsible for the increased *RPL9* expression in RTT (Figure S18, Additional file 1). It should, however, be noted that changes in isoform usage cannot only be attributed to alternative splicing but can also be the result of alternative promotor usage or alternative polyadenylation [44].

In order to determine whether the differential expressed processes are direct regulatory targets of MeCP2, we re-analyzed the MeCP2 ChIP-Seq data of Rube et al. (GSE71126 [31]). In the original study, Rube et al. measured MeCP2 chromatin binding in mouse olfactory neuroepithelia at higher resolution than other available animal and human MeCP2 ChIP-seq data. We found significantly higher MeCP2 enrichment around the transcription start site (i.e., TSS +/- 3000 bp) of particularly neuronal migration and development-related genes (blue cluster) than would have been expected by chance (Fig. 4B). For instance, *Ameboidal-type cell migration* – cell migration using temporary cellular projections – might be under direct control by MeCP2 as it reached high levels of statistical significance at protein, RNA, and MeCP2 promotor-binding levels (Figs. 3 and 4, and S19, Additional file 1). In contrast, the observed transcriptomics and proteomics changes in *Cytoplasmic translation* and *RNA splicing*,* via transesterification reactions* (Figs. 3 and 4A) level may not have directly resulted from changes in MeCP2 promotor-binding activity (Figs. 4B and S19, Additional file 1).

Because the active X chromosome is different between the RTT and IC samples, we excluded X-linked genes from our organoid analyses. To nevertheless assess whether X chromosomal alterations could play a role in RTT pathology, we performed differential gene expression analysis of X-linked genes in independent RNA-seq validation datasets from post-mortem brains (GSE128380) and male interneurons (GSE117511). The X-linked genes *MAOA* and *IL13RA* were differentially expressed (P value < 0.05) in both datasets (Figure S20, Additional file 1).

### Network inference identified differentially expressed proteins and lncRNAs as key regulators

**Fig. 5:**
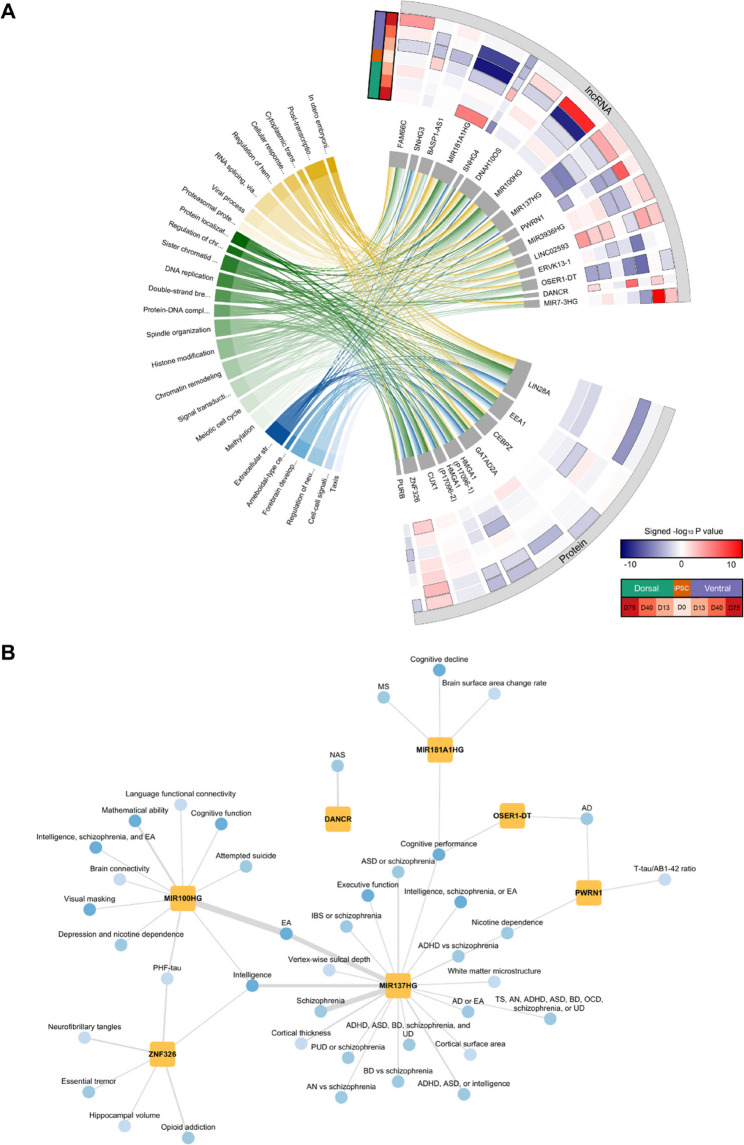
Identification of potential regulators. **A** The chord diagram shows the potential lncRNA and protein regulators (right) for each of the 26 GO-BP terms (left). The heatmap on the right visualizes the spatiotemporal differential expression (signed -log_10_ P value) profile for each of the potential regulators. The black border around the heatmap tiles indicates an FDR-adjusted P value < 0.05. **B** Network diagram of the association between the potential regulators and CNS-related traits (GWAS Catalog version 2023-12-20). The potential regulators from panel A were included in the diagram if they were differentially expressed at minimally three time points and/or brain regions and had minimally one association with a CNS-related trait. AD: Alzheimer’s disease; ADHD: attention deficit hyperactivity disorder; AN: anorexia nervosa; ASD: autism spectrum disorder; BD: bipolar disorder; EA: education attainment; MS: multiple sclerosis; NAS: neonatal abstinence syndrome, OCD: obsessive-compulsive disorder; PUD: peptic ulcer disease; TS: Tourette syndrome; UD: unipolar depression

The altered expression profile of the identified biological processes may either have resulted directly from the loss of MeCP2 binding to the gene promoter or relate to changes in the expression of other protein or lncRNA regulators. To identify potential lncRNA and protein regulators of the 26 GO-BP terms, we performed network inference with the eigengene (i.e., the first principal component) of the GO-BP terms as target variables. This analysis resulted in a network with 15 candidate lncRNA regulators and 9 protein regulators (Fig. 5A). Of these potential regulators, 11 lncRNAs and 2 proteins were differentially expressed (i.e., FDR-adjusted P value < 0.05) at minimally three time points and/or brain region (Fig. 5A). Among these differentially expressed regulators, the transcription factor ZNF326 as well as several lncRNA genes, such as *PWRN1*, *OSER1-DT*, and the miRNA host genes *MIR100HG*, *MIR137HG*, and *MIR181A1HG*, are associated with multiple CNS-related traits (GWAS Catalog [45] version 2023-12-20) (Fig. 5B). Together, the convergence of (1) predicted regulation of RTT-dysregulated processes, (2) consistent temporal and/or regional dysregulation, and (3) independent genetic links to CNS-related traits supports their potential relevance to RTT pathophysiology.

### Imprinted genes are more likely to be differentially expressed in RTT

**Fig. 6:**
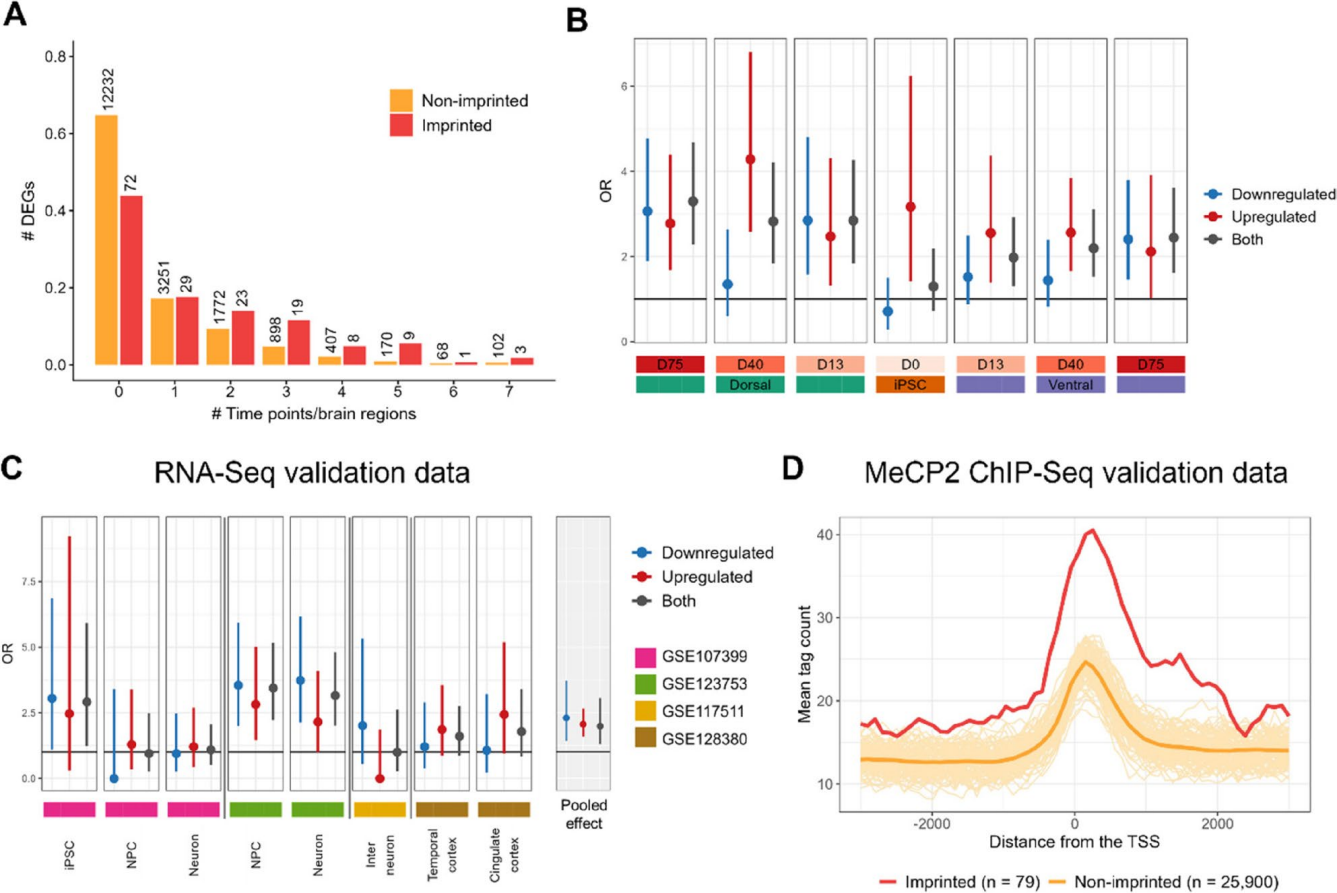
RTT-specific alterations in imprinted genes. **A** A bar chart visualizing the distribution of the number of imprinted and non-imprinted genes that are differentially expressed (i.e., differentially expressed genes; DEGs) at different numbers of time points/brain regions. The absolute number of genes is indicated by the text on top of the bars. In this plot, it can be seen that three imprinted genes (i.e., MEG3, MEG8, and ZNF229) are differentially expressed at all seven time points and/or brain regions. A gene was considered differentially expressed in case of a |log_2_FC| > 1 and FDR-adjusted P value < 0.05. **B** The odds ratio (OR) and the corresponding 95% confidence interval of being downregulated (blue), upregulated (red), or differentially expressed in either direction (grey) for imprinted genes versus non-imprinted genes is shown at the different time points and brain regions. For most time points and brain regions, the OR is significantly higher than one (i.e., black horizontal line). **C** The odds ratio (OR) and the corresponding 95% confidence interval of being downregulated (blue), upregulated (red), or differentially expressed in either direction (grey) for imprinted genes versus non-imprinted genes is shown for the different RNA-seq validation sets. Note that for the validation sets the nominal P value instead of the adjusted P value was used to determine the differential expression status of a gene to increase the number of DEGs for the statistical analysis (i.e., Fisher’s exact test). **D** Mean tag counts at the promoter region (+/- 3000 bp from the TSS) for imprinted (*n* = 79) and non-imprinted genes (*n* = 25,000) in the publicly available MeCP2 ChIP-Seq data (GSE71126). The orange lines on the background are the mean tag counts of 100 random permutations where for each permutation 79 random genes were selected. These permutations indicated that MeCP2 binds more abundantly to the promoter region of imprinted genes than expected for a random set of genes

The involvement of dysregulated genomic imprinting in RTT pathology is under debate. While some studies show a dysregulated imprinted status of several genes in response to MeCP2 loss-of-function [35, 46], others show that the imprinted status remains unaffected [47]. To investigate the potential pathological role of imprinted genes in RTT, we characterized the differential expression profile of previously reported or predicted imprinted genes (*geneimprint.com*). Only 1% of the genes that passed QC were predicted or reported to be imprinted (i.e., N_imprinted_ = 164). Interestingly, imprinted genes tend to be differentially expressed at more time points and/or brain regions than non-imprinted genes (Fig. 6A). For instance, the imprinted genes *MEG3*, *MEG8*, *ZNF229*, and *MEST* genes were significantly upregulated at minimally six of the seven time points/brain regions (Figure S21, Additional file 1). Notably, *MEST* has two promoter regions of which only one is imprinted [48]. The *MEST* isoform transcribed from the imprinted promoter (e.g.,* MEST-201*) demonstrated a significant increase in the relative isoform abundance in RTT, while the *MEST* isoform transcribed from the non-imprinted promoter (e.g.,* MEST-202*) had a decreased relative isoform abundance (Figure S22, Additional file 1), suggesting that the increased *MEST* mRNA levels are particularly driven by increased activity of the imprinted promoter in RTT.

In general, we found that at almost all time points and brain regions, the odds of an imprinted gene to be differentially expressed was significantly higher than for the non-imprinted genes (Fig. 6B), a property that was also observed in multiple validation sets (Fig. 6C). Furthermore, the re-analysis of publicly available MeCP2 ChIP-Seq data (GSE71126 [31]) indicated that MeCP2 binds more abundantly to the promoter region of imprinted genes than non-imprinted genes (P value = 8.9e-9, Fig. 6D). Imprinted genes with higher levels of MeCP2 promoter enrichment also exhibited larger expression changes in MeCP2 knockout mice (Figure S23, Additional file 1). Hence, the direct binding of MeCP2 to imprinted promoter regions might be one way by which MeCP2 can regulate the expression of imprinted genes. Interestingly, from the 26 identified GO terms, only *Cell-cell signaling by wnt (GO:0198738)* is overrepresented with imprinted genes (FDR-adjusted P value = 8.7e-3).

To investigate whether the changes in the expression profiles of imprinted genes are associated with the loss or gain of bi-allelic expression, we performed allele-specific expression (ASE) analysis on our transcriptomics dataset. It should be noted that due to the developmental stage- and tissue-specific nature of genomic imprinting [49], not all of the previously reported imprinted genes are expected to have a mono-allelic expression in the developing forebrain organoids. Indeed, ASE analysis on our transcriptomics dataset revealed that at least 11 of the reported imprinted genes had a bi-allelic expression in at least four time points and/or brain regions in the IC brain organoids (Table S2, Additional file 1). Interestingly, four of these genes (*MZF1*,* OBSCN*,* TSHZ3*, and *RHOBTB3*) lost their bi-allelic expression in RTT (i.e., Mann-Whitney U Test P value < 0.1 at minimally four time points and/or brain regions) (Figure S24A, Additional file 1). We also identified two imprinted genes (*MAGI2* and *DLK1*) with a monoallelic expression in IC that exhibited a bi-allelic expression in the RTT organoids (Figure S24A, Additional file 1). Nevertheless, the loss- or gain- of bi-allelic expression was mostly not associated with a decrease or increase in total expression levels (Figure S24B, Additional file 1). Only the *MEG3*,* MEG8* and *MEG9* genes located in the same genomic region as the *DLK1* gene, an imprinted gene that lost its mono-allelic expression in the RTT organoids, demonstrated strong upregulation in RTT. However, as these imprinted genes demonstrated a fold change increase of significantly larger than two (Figure S25, Additional file 1), the increase cannot solely be attributed to the gain of bi-allelic expression.

## Discussion

In the present study, region-specific forebrain organoids with an RTT-causing MeCP2 mutation (MeCP2:R255X, rs61749721) and its isogenic control (IC) were utilized to characterize the spatiotemporal alterations in the RTT transcriptomic and proteomic profile. While GABAergic signaling was found to be specifically altered at the latest neurodevelopmental stages, transcriptomic alterations in neuronal migration and development, DNA-associated processes, and post-transcriptional regulation occurred across multiple developmental phases. The involvement of these biological processes in RTT pathology was also observed at the protein level and in multiple independent RNA-seq datasets. Finally, we proposed potential lncRNA and protein regulators of these processes and provided evidence for a pathological role of imprinted genes.

### A role of MeCP2 in chromatin remodeling and genomic imprinting

At both transcript and protein levels, we identified several DNA-associated processes that were significantly enriched with upregulated genes across multiple time points and regions (Figs. 3, green cluster). While the direct role of MeCP2 in the formation of heterochromatin compartments has recently been challenged [50], our findings suggest that MeCP2 may instead play an indirect role in modulating chromatin state by regulating the expression of genes involved in DNA-associated processes, such as *Protein-DNA complex assembly*, *DNA replication*, and *Chromatin remodeling.* Notably, these DNA-associated processes were also found enriched in the validation sets with varying tissue/cell types, but with contrasting directions of effect (Fig. 4A). Opposing directions of effect at the gene level have previously been observed among different cell/tissue types in RTT [51, 52]. Given the dual role of MeCP2 as a transcriptional activator and repressor [53], the direction of MeCP2 mutations-associated changes may depend on the biological context, such as mutation type, cell type, and genetic background.

Among others, chromatin remodeling is essential for regulating a gene’s imprinted status [54]. Importantly, in addition to the dysregulated expression of chromatin remodeling-associated genes, we found that imprinted genes are more likely to be differentially expressed (both up- and downregulated) compared to non-imprinted genes (Fig. 6A-B). This pattern was consistent across several independent validation sets (Fig. 6C). Since the imprinting status might not be completely faithful in iPSC-derived model systems [55, 56], RNA-seq data (Fig. 6C, GSE128380) and ChIP-seq data (Fig. 6D) from non-iPSC-derived model systems were included among the validation sets. Despite the potential unfaithful imprinting status, iPSC-derived model systems still exhibited preference for differential expression of imprinted genes (Fig. 6B-C). In such case, this observation may be explained by previous research indicating that MeCP2 binding to DNA is largely independent of DNA methylation status [31].

Although the role of MeCP2 in genomic imprinting is debated [47], due to *Dlx5* loss-of-imprinting in *Mecp2*-null mice, MeCP2 has previously been proposed as a regulator of imprinting [35, 46]. Moreover, the upregulation of the murine homolog of *DLX6* [46], *MEG3* [57], and miRNA genes in the *MEG3*-imprinted region [58] (i.e., 14q32.2 in human) has also been observed in RTT mice models. The role of MeCP2 in imprinting has been further elucidated by Kernohan et al., who propose that MeCP2 can cooperate with ATRX and cohesion to silence several imprinted genes in mouse brains [59]. In line with this, we found that MeCP2 indeed preferentially binds to the promoter region of imprinted genes (Fig. 6D). In addition to transcriptional repression, MeCP2 binding to a promoter can also lead to the recruitment of transcriptional activators and the subsequent positive regulation of the gene expression [53]. A similar mechanism may be at play at the promoter of imprinted genes that exhibited a downregulated expression in RTT. However, we found no conclusive evidence for the occurrence of the gain or loss of bi-allelic gene expression of imprinted genes in RTT (Figures S24 and S25, Additional file 1). Taken together, although previous studies pointed towards the involvement of imprinted genes in RTT pathology, our analysis shows, for the first time, systematic transcriptomic changes of imprinted genes across multiple neurodevelopmental stages and validation datasets.

Imprinted genes have been linked to various neurodevelopmental disorders, like Angelman, Prader-Willi, Temple, and Kagami-Ogata syndromes [60, 61]. Specifically, Temple and Kagami-Ogata syndromes affect the chromosome 14q32 imprinted locus, the same genomic location where we found the loss of mono-allelic expression of the *DLK1* gene and upregulated expression levels of the *MEG3*, *MEG8*, and *MEG9* genes (Figure S24, Additional file 1). Interestingly, Temple and Kagami-Ogata syndromes have similar developmental manifestations as RTT, including hypotonia, motor delay, speech delay, and intellectual disability [62], possibly pointing towards similar molecular underpinnings. Prader-Willi syndrome, another neurodevelopmental disorder, has also been associated with increased gene expression levels of the imprinted *MEG* family in iPSCs [63]. These findings, together, suggest a possible involvement of the chromosome 14q32 imprinted locus in RTT and other neurodevelopmental disorders.

Among the identified potential regulators of the DNA-associated processes was the *Prader-Willi Region Non-Protein Coding RNA 1 (PWRN1)* gene, which was differentially expressed at four time points and/or brain regions (Fig. 5). Although *PWRN1* itself is not classified as imprinted (*geneimprint.com*), it resides in the imprinted Prader-Willi syndrome region on chromosome 15 and is known to be mono-allelically expressed in the brain [64]. The exact functional role of *PWRN1* is unknown, but it has been shown to inhibit cellular proliferation [65, 66], a process that requires extensive chromatin remodeling [67] and is altered in MeCP2 loss-of-function conditions [68]. Besides *PWRN1*, some other identified potential regulators with a differential expression profile are also located in the genomic regions (< 500 kb) of predicted or reported imprinted genes, including *LINC02593* (neighboring imprinted gene: *DVL1*), *OSER1-DT* (neighboring imprinted gene: *GDAP1L1*), and *MIR3936HG* (neighboring imprinted gene: *CSF2*).

### Impaired neuronal development and function in RTT

We showed that several GO terms related to neuronal development and migration were significantly enriched with either up- or downregulated genes at multiple time points and brain regions (Fig. 3, blue cluster). Specifically, among the enriched GO terms, processes such as Wnt signaling [69], the organization of extracellular matrix [70], the formation of neural projections [71, 72], as well as taxis and ameboidal-type cell migration, are known to play an important role in the initiation and/or progression of neuronal migration. This is supported by previous studies that identified neuronal differentiation and migration defects in RTT brain organoids [14, 73]. Furthermore, we also found many of these processes to be significantly enriched in multiple validation sets (Fig. 4, blue cluster), further confirming our findings.

At days 40 and 75, when the organoids resemble the human fetal brain during the first and second trimesters [74], we observed differential expression of neuronal proteins (Fig. 2C), further highlighting the presence of neuronal dysfunction. These neuronal proteins include neurofilaments NEFL and NEFM [75], SNCG [76], LY6H – a regulator of acetylcholine receptor activity [77], NTN1 – a regulator of axon guidance and neuroprotection [78], and VGF – a regulator of neurogenesis and neuroplasticity [79]. Besides, we also observed an upregulation of GABAergic signaling-related genes at the latest developmental stages in the ventral region forebrain organoids (Fig. 2D). Indeed, GABAergic signaling has been described to play a central role in the pathogenesis of RTT and several other neurodevelopmental disorders [80]. Particularly, restoring *Mecp2* expression in GABAergic neurons could rescue RTT-associated symptoms in mice [81] and mutations in the GABA-B receptor R2 (GABBR2) have been associated with RTT-like phenotypes [82]. Nevertheless, in contrast to the enhanced expression of GABAergic signalling-associated genes in our results, previous studies identified reduced GABAergic signalling activity and lower levels of Gad1 and Gad2 in RTT mice models [81, 83]. However, GABAergic signalling is known to be a dynamic process that changes throughout the different pre- and post-natal development stages [84]. Hence, the GABAergic signalling-related changes observed in RTT do not remain stable, but instead fluctuate during neuronal development, possibly explaining discrepancies between our findings and the literature. Together, our study indicates that dysfunctions in synaptic transmission in RTT can already manifest as early as during prenatal neurodevelopment.

HMGA1, a known regulator of chromatin structure and gene transcription [85], is one of the predicted protein regulators of the neural development- and migration-related processes (Fig. 5A). Interestingly, this protein has previously been found to control the development and migration of neural crest cells via the Wnt pathway [86]. The reduced protein expression of HMGA1 in the RTT organoids might be responsible for the aberrations in neuronal migration. Other potential lncRNA and protein regulators, such as ZNF326, *MIR100HG*, *MIR137HG*, and *MIR181A1HG*, were also shown to have altered expression profiles in RTT (Fig. 5A) and are associated with one or more CNS-related traits (Fig. 5B), suggesting their importance in neuronal function. Similarly, the upregulated expression of the lncRNA regulator *LINC02593* has previously been found to be associated with autism spectrum disorders [87]. These lncRNAs and proteins, as central regulators of multiple RTT-affected biological processes, may offer promising therapeutic opportunities. For instance, regulators such as IGF-1 and SIGMAR, have already revealed favourable results as RTT therapeutic targets [88, 89].

### RNA splicing and translation defects in RTT pathology

Also at the post-transcriptional level, we identified alterations in the RTT forebrain organoids. These alterations were particularly related to post-transcriptional regulation of gene expression, cytoplasmic translation, and RNA splicing events (Figs. 3 and 4). The latter two processes were, however, not associated with enhanced MeCP2 binding to the genes’ promoter regions (Fig. 4B).

Regarding cytoplasmic translation, in our model system, there was a slight increase in the protein and RNA expression of ribosomal protein-encoding genes in the dorsal/glutamatergic region, whereas there was a more pronounced decrease at the latest time points of the ventral/GABAergic region (Figures S15 and S16, Additional file 1). A decrease in the global translational capacity in RTT neurons has previously been observed [5]. However, these neuronal cell cultures included both GABAergic and glutamatergic neurons. Hence, the decreased global translation observed by Rodrigues et al. may have been caused by the strong downregulation of global translation in the GABAergic neurons that potentially masked the (smaller) translational upregulation in the glutamatergic neurons. The involvement of aberrant cytoplasmic translation in RTT is further emphasized by the fact that mutations in many mRNA translation-associated genes are associated with microcephaly [90], a common clinical characteristic of RTT patients [91].

In addition to the differential expression of ribosomal proteins, we also observed changes in the expression of RNA splicing-related genes (Figs. 3 and 4). Alternative splicing, together with alternative promotor usage and alternative polyadenylation, drives alternative transcript isoform usage in a cell [44]. Consistent with the identified expression alterations of RNA splicing-related genes, several genes and processes with an altered expression profile in RTT also showed changes in their relative isoform expression levels in RTT. Differential alternative isoform usage was found to particularly occur for genes involved in cytoplasmic translation (Figure S17, Additional file 1). Differential splicing of such genes has previously been suggested to be a cellular coping mechanism to deal with stress [92], a well-established hallmark of RTT [28]. Indeed, alterations in alternative splicing events have been described in RTT mouse models [93, 94], suggesting a role of alternative isoform usage in the pathology of RTT.

### Limitations

It is important to note that the RTT and IC brain organoids were generated from a single RTT patient. To address this limitation and other potential limitations of the current experimental approach (e.g., dissociation of the organoids with Accutase before lysis), we validated our findings in multiple independent cohorts which included RNA-seq, proteomics, and ChIP-seq data from different genetic backgrounds, RTT-causing *MECP2* mutations, and cell types/tissues. This validation confirmed that the identified RTT-associated GO terms were not specific to our study but could be observed across experimental setups, *MECP2* mutations, and genetic backgrounds. Furthermore, the potential unfaithful imprinting status in iPSC-derived model systems was addressed by validating our findings in RNA-seq (Fig. 6C, GSE128380) and ChIP-seq data (Fig. 6D) from non-iPSC-derived model systems. While the distinct study designs and model systems of these validation datasets highlight the generalizability of our findings, they do not allow for the validation of the alterations that are specific to a brain region or developmental time point.

## Conclusions

By collecting and analyzing transcriptomics data from spatiotemporal RTT and IC forebrain organoid models, our study identified expression alterations in processes related to chromatin remodeling and genomic imprinting, post-transcriptional regulation, and neuronal development and functioning. Especially the identification of transcriptomic changes in GABAergic signaling during early neurodevelopment, along with the systematic alterations in imprinted genes represent novel findings that may open new avenues for therapeutic interventions. Nevertheless, the future collection and analysis of iPSC-derived brain organoids from patients with other RTT-causing *MECP2* mutations will be needed to distinguish between common and patient/mutation-specific RTT alterations. Furthermore, future studies are needed to functionally validate the significance of the identified processes and molecular targets in RTT pathogenesis, as well as their value as therapeutic targets. 

## Supplementary Information

Additional file 1.

## Data Availability

The proteomics data have been deposited in the MassIVE repository with the dataset identifier MSV000095738. The processed RNA-seq data (i.e., raw count matrix) have been deposited on Zenodo (10.5281/zenodo.13383211). The R scripts for the analysis of the datasets are available on GitHub (https://github.com/SyNUM-lab/OrganoidAnalysis).

## Abbreviations

ASE: Allele-specific expression
CPM: Counts per million
DEG: Differentially expressed gene
FDR: False discovery rate
GEO: Gene Expression Omnibus
GO(-BP): Gene ontology(-biological process)
GSEA: Gene set enrichment analysis
IC: Isogenic control
iPSC: Induced pluripotent stem cell
LC-MS: Liquid chromatography-mass spectrometry
log2FC: log2 fold change
MECP2: Methyl-CpG-binding protein 2
MNAR: Missing not at random
NPC: Neural progenitor cell
OR: Odds ratio
PCA: Principal component analysis
RTT: Rett syndrome
SNP: Single nucleotide polymorphisms
TSS: Transcription start site

## References

[1] Banerjee A, Miller MT, Li K, Sur M, Kaufmann WE. Towards a better diagnosis and treatment of Rett syndrome: a model synaptic disorder. Brain. 2019;142(2):239–48. 30649225 10.1093/brain/awy323 PMC6933507

[2] Amir RE, Van den Veyver IB, Wan M, Tran CQ, Francke U, Zoghbi HY. Rett syndrome is caused by mutations in X-linked MECP2, encoding methyl-CpG-binding protein 2. Nat Genet. 1999;23(2):185–8. 10508514 10.1038/13810

[3] Bin Akhtar G, Buist M, Rastegar M. MeCP2 and transcriptional control of eukaryotic gene expression. Eur J Cell Biol. 2022;101(3):151237. 35588541 10.1016/j.ejcb.2022.151237

[4] Cheng TL, Chen J, Wan H, Tang B, Tian W, Liao L, et al. Regulation of mRNA splicing by MeCP2 via epigenetic modifications in the brain. Sci Rep. 2017;7:42790. 28211484 10.1038/srep42790 PMC5314398

[5] Rodrigues DC, Mufteev M, Weatheritt RJ, Djuric U, Ha KCH, Ross PJ, et al. Shifts in ribosome engagement impact key gene sets in neurodevelopment and ubiquitination in Rett syndrome. Cell Rep. 2020;30(12):4179–e9611. 32209477 10.1016/j.celrep.2020.02.107

[6] Zlatic SA, Duong D, Gadalla KKE, Murage B, Ping L, Shah R, et al. Convergent cerebrospinal fluid proteomes and metabolic ontologies in humans and animal models of Rett syndrome. iScience. 2022;25(9):104966. 36060065 10.1016/j.isci.2022.104966 PMC9437849

[7] Tomasello DL, Barrasa MI, Mankus D, Alarcon KI, Lytton-Jean AKR, Liu XS, et al. Mitochondrial dysfunction and increased reactive oxygen species production in MECP2 mutant astrocytes and their impact on neurons. Sci Rep. 2024;14(1):20565. 39232000 10.1038/s41598-024-71040-y PMC11374804

[8] Samarasinghe RA, Miranda OA, Buth JE, Mitchell S, Ferando I, Watanabe M, et al. Identification of neural oscillations and epileptiform changes in human brain organoids. Nat Neurosci. 2021;24(10):1488–500. 34426698 10.1038/s41593-021-00906-5 PMC9070733

[9] Xiang Y, Tanaka Y, Patterson B, Hwang SM, Hysolli E, Cakir B, et al. Dysregulation of BRD4 function underlies the functional abnormalities of MeCP2 mutant neurons. Mol Cell. 2020;79(1):84–e989. 32526163 10.1016/j.molcel.2020.05.016 PMC7375197

[10] Grenier K, Kao J, Diamandis P. Three-dimensional modeling of human neurodegeneration: brain organoids coming of age. Mol Psychiatry. 2020;25(2):254–74. 31444473 10.1038/s41380-019-0500-7

[11] Adams JW, Cugola FR, Muotri AR. Brain organoids as tools for modeling human neurodevelopmental disorders. Physiology. 2019;34(5):365–75. 31389776 10.1152/physiol.00005.2019 PMC6863377

[12] Wither RG, Lang M, Zhang L, Eubanks JH. Regional MeCP2 expression levels in the female MeCP2-deficient mouse brain correlate with specific behavioral impairments. Exp Neurol. 2013;239:49–59. 23022455 10.1016/j.expneurol.2012.09.005

[13] D’Mello SR 3. MECP2 and the biology of MECP2 duplication syndrome. J Neurochem. 2021;159(1):29–60. 33638179 10.1111/jnc.15331

[14] Gomes AR, Fernandes TG, Vaz SH, Silva TP, Bekman EP, Xapelli S, et al. Modeling Rett syndrome with human patient-specific forebrain organoids. Front Cell Dev Biol. 2020;8:610427. 33363173 10.3389/fcell.2020.610427 PMC7758289

[15] Bahram Sangani N, Koetsier J, Gomes AR, Diogo MM, Fernandes TG, Bouwman FG, et al. Involvement of extracellular vesicle microRNA clusters in developing healthy and Rett syndrome brain organoids. Cell Mol Life Sci. 2024;81(1):410. 39305343 10.1007/s00018-024-05409-7 PMC11416455

[16] Martin M. Cutadapt removes adapter sequences from high-throughput sequencing reads. EMBnet J. 2011;17(1):10–2.

[17] Andrews S. FastQC: a quality control tool for high throughput sequence data. Cambridge, United Kingdom: babraham bioinformatics, babraham institute; 2010.

[18] Langmead B, Salzberg SL. Fast gapped-read alignment with Bowtie 2. Nat Methods. 2012;9(4):357–9. 22388286 10.1038/nmeth.1923 PMC3322381

[19] Li B, Dewey CN. RSEM: accurate transcript quantification from RNA-Seq data with or without a reference genome. BMC Bioinformatics. 2011;12:323. 21816040 10.1186/1471-2105-12-323 PMC3163565

[20] Robinson MD, McCarthy DJ, Smyth GK. edgeR: a Bioconductor package for differential expression analysis of digital gene expression data. Bioinformatics. 2010;26(1):139–40. 19910308 10.1093/bioinformatics/btp616 PMC2796818

[21] Zhang X, Smits AH, van Tilburg GB, Ovaa H, Huber W, Vermeulen M. Proteome-wide identification of ubiquitin interactions using UbIA-MS. Nat Protoc. 2018;13(3):530–50. 29446774 10.1038/nprot.2017.147

[22] Durinck S, Spellman PT, Birney E, Huber W. Mapping identifiers for the integration of genomic datasets with the R/Bioconductor package biomaRt. Nat Protoc. 2009;4(8):1184–91. 19617889 10.1038/nprot.2009.97 PMC3159387

[23] Wu T, Hu E, Xu S, Chen M, Guo P, Dai Z, et al. clusterProfiler 4.0: A universal enrichment tool for interpreting omics data. Innov (Camb). 2021;2(3):100141. 10.1016/j.xinn.2021.100141 PMC8454663 34557778

[24] Sayols S. rrvgo: a Bioconductor package for interpreting lists of gene ontology terms. MicroPubl Biol. 2023. 10.17912/micropub.biology.000811 PMC10155054 37151216

[25] Ritchie ME, Phipson B, Wu D, Hu Y, Law CW, Shi W, et al. limma powers differential expression analyses for RNA-sequencing and microarray studies. Nucleic Acids Res. 2015;43(7):e47. 25605792 10.1093/nar/gkv007 PMC4402510

[26] Agrawal A, Balcı H, Hanspers K, Coort SL, Martens M, Slenter DN et al. WikiPathways 2024: next generation pathway database. Nucleic Acids Res. 2023;52(D1):D679-D689. 10.1093/nar/gkad960 PMC10767877 37941138

[27] Barrett T, Wilhite SE, Ledoux P, Evangelista C, Kim IF, Tomashevsky M, et al. NCBI GEO: archive for functional genomics data sets–update. Nucleic Acids Res. 2013;41(Database issue):D991–5. 23193258 10.1093/nar/gks1193 PMC3531084

[28] Ohashi M, Korsakova E, Allen D, Lee P, Fu K, Vargas BS, et al. Loss of MECP2 Leads to Activation of P53 and Neuronal Senescence. Stem Cell Rep. 2018;10(5):1453–63. 10.1016/j.stemcr.2018.04.001 PMC5995366 29742391

[29] Aldinger KA, Timms AE, MacDonald JW, McNamara HK, Herstein JS, Bammler TK, et al. Transcriptome data of temporal and cingulate cortex in the Rett syndrome brain. Sci Data. 2020;7(1):192. 32561870 10.1038/s41597-020-0527-2 PMC7305197

[30] Varderidou-Minasian S, Hinz L, Hagemans D, Posthuma D, Altelaar M, Heine VM. Quantitative proteomic analysis of Rett iPSC-derived neuronal progenitors. Mol Autism. 2020;11(1):38. 32460858 10.1186/s13229-020-00344-3 PMC7251722

[31] Rube HT, Lee W, Hejna M, Chen H, Yasui DH, Hess JF, et al. Sequence features accurately predict genome-wide MeCP2 binding in vivo. Nat Commun. 2016;7:11025. 27008915 10.1038/ncomms11025 PMC4820824

[32] Huynh-Thu VA, Irrthum A, Wehenkel L, Geurts P. Inferring regulatory networks from expression data using tree-based methods. PLoS ONE. 2010;5(9):e12776. 10.1371/journal.pone.0012776 PMC2946910 20927193

[33] Lambert SA, Jolma A, Campitelli LF, Das PK, Yin Y, Albu M, et al. Hum Transcription Factors Cell. 2018;172(4):650–65. 10.1016/j.cell.2018.01.029 PMC12908702 29425488

[34] Zheng Y, Luo H, Teng X, Hao X, Yan X, Tang Y, et al. NPInter v5.0: ncRNA interaction database in a new era. Nucleic Acids Res. 2023;51(D1):D232–9. 36373614 10.1093/nar/gkac1002 PMC9825547

[35] Pescucci C, Meloni I, Renieri A. Is Rett syndrome a loss-of-imprinting disorder? Nat Genet. 2005;37(1):10–1. 15624014 10.1038/ng0105-10

[36] Balduzzi S, Rücker G, Schwarzer G. How to perform a meta-analysis with R: a practical tutorial. Evid Based Ment Health. 2019;22(4):153–60. 31563865 10.1136/ebmental-2019-300117 PMC10231495

[37] Van der Auwera GA, O’Connor BD. Genomics in the cloud: using Docker, GATK, and WDL in Terra. O’Reilly Media; 2020.

[38] van Beek D, Verdonschot J, Derks K, Brunner H, de Kok TM, Arts ICW, et al. Allele-specific expression analysis for complex genetic phenotypes applied to a unique dilated cardiomyopathy cohort. Sci Rep. 2023;13(1):564. 36631531 10.1038/s41598-023-27591-7 PMC9834222

[39] Keane TM, Goodstadt L, Danecek P, White MA, Wong K, Yalcin B, et al. Mouse genomic variation and its effect on phenotypes and gene regulation. Nature. 2011;477(7364):289–94. 21921910 10.1038/nature10413 PMC3276836

[40] Burke EE, Chenoweth JG, Shin JH, Collado-Torres L, Kim SK, Micali N, et al. Dissecting transcriptomic signatures of neuronal differentiation and maturation using iPSCs. Nat Commun. 2020;11(1):462. 31974374 10.1038/s41467-019-14266-z PMC6978526

[41] Uhlén M, Fagerberg L, Hallström BM, Lindskog C, Oksvold P, Mardinoglu A, et al. Proteomics. Tissue-based map of the human proteome. Science. 2015;347(6220):1260419. 25613900 10.1126/science.1260419

[42] Bond AM, Vangompel MJ, Sametsky EA, Clark MF, Savage JC, Disterhoft JF, et al. Balanced gene regulation by an embryonic brain ncRNA is critical for adult hippocampal GABA circuitry. Nat Neurosci. 2009;12(8):1020–7. 19620975 10.1038/nn.2371 PMC3203213

[43] Lund IV, Hu Y, Raol YH, Benham RS, Faris R, Russek SJ, et al. BDNF selectively regulates GABAA receptor transcription by activation of the JAK/STAT pathway. Sci Signal. 2008;1(41):ra9. 18922788 10.1126/scisignal.1162396 PMC2651003

[44] Anvar SY, Allard G, Tseng E, Sheynkman GM, de Klerk E, Vermaat M, et al. Full-length mRNA sequencing uncovers a widespread coupling between transcription initiation and mRNA processing. Genome Biol. 2018;19(1):46. 29598823 10.1186/s13059-018-1418-0 PMC5877393

[45] Sollis E, Mosaku A, Abid A, Buniello A, Cerezo M, Gil L, et al. The NHGRI-EBI GWAS Catalog: knowledgebase and deposition resource. Nucleic Acids Res. 2023;51(D1):D977–85. 36350656 10.1093/nar/gkac1010 PMC9825413

[46] Horike S, Cai S, Miyano M, Cheng JF, Kohwi-Shigematsu T. Loss of silent-chromatin looping and impaired imprinting of DLX5 in Rett syndrome. Nat Genet. 2005;37(1):31–40. 15608638 10.1038/ng1491

[47] Schüle B, Li HH, Fisch-Kohl C, Purmann C, Francke U. DLX5 and DLX6 expression is biallelic and not modulated by MeCP2 deficiency. Am J Hum Genet. 2007;81(3):492–506. 17701895 10.1086/520063 PMC1950824

[48] Kosaki K, Kosaki R, Craigen WJ, Matsuo N. Isoform-specific imprinting of the human PEG1/MEST gene. Am J Hum Genet. 2000;66(1):309–12. 10631159 10.1086/302712 PMC1288335

[49] Babak T, DeVeale B, Tsang EK, Zhou Y, Li X, Smith KS, et al. Genetic conflict reflected in tissue-specific maps of genomic imprinting in human and mouse. Nat Genet. 2015;47(5):544–9. 25848752 10.1038/ng.3274 PMC4414907

[50] Pantier R, Brown M, Han S, Paton K, Meek S, Montavon T, et al. MeCP2 binds to methylated DNA independently of phase separation and heterochromatin organisation. Nat Commun. 2024;15(1):3880. 38719804 10.1038/s41467-024-47395-1 PMC11079052

[51] Ehrhart F, Coort SL, Eijssen L, Cirillo E, Smeets EE, Bahram Sangani N, et al. Integrated analysis of human transcriptome data for Rett syndrome finds a network of involved genes. World J Biol Psychiatry. 2020;21(10):712–25. 30907210 10.1080/15622975.2019.1593501

[52] Haase F, Singh R, Gloss B, Tam P, Gold W. Meta-Analysis Identifies BDNF and Novel Common Genes Differently Altered in Cross-Species Models of Rett Syndrome. Int J Mol Sci. 2022;23:19. 10.3390/ijms231911125 PMC9570315 36232428

[53] Tillotson R, Bird A. The Molecular Basis of MeCP2 Function in the Brain. J Mol Biol. 2020;432(6):1602–23. 31629770 10.1016/j.jmb.2019.10.004

[54] Wang SE, Jiang YH. Novel epigenetic molecular therapies for imprinting disorders. Mol Psychiatry. 2023;28(8):3182–93. 37626134 10.1038/s41380-023-02208-7 PMC10618104

[55] Pham A, Selenou C, Giabicani E, Fontaine V, Marteau S, Brioude F, et al. Maintenance of methylation profile in imprinting control regions in human induced pluripotent stem cells. Clin Epigenetics. 2022;14(1):190. 36578048 10.1186/s13148-022-01410-8 PMC9798676

[56] Bar S, Schachter M, Eldar-Geva T, Benvenisty N. Large-Scale Analysis of Loss of Imprinting in Human Pluripotent Stem Cells. Cell Rep. 2017;19(5):957–68. 28467909 10.1016/j.celrep.2017.04.020

[57] Jordan C, Li HH, Kwan HC, Francke U. Cerebellar gene expression profiles of mouse models for Rett syndrome reveal novel MeCP2 targets. BMC Med Genet. 2007;8:36. 17584923 10.1186/1471-2350-8-36 PMC1931432

[58] Wu H, Tao J, Chen PJ, Shahab A, Ge W, Hart RP, et al. Genome-wide analysis reveals methyl-CpG-binding protein 2-dependent regulation of microRNAs in a mouse model of Rett syndrome. Proc Natl Acad Sci U S A. 2010;107(42):18161–6. 20921386 10.1073/pnas.1005595107 PMC2964235

[59] Kernohan KD, Jiang Y, Tremblay DC, Bonvissuto AC, Eubanks JH, Mann MR, et al. ATRX partners with cohesin and MeCP2 and contributes to developmental silencing of imprinted genes in the brain. Dev Cell. 2010;18(2):191–202. 20159591 10.1016/j.devcel.2009.12.017

[60] Isles AR. The contribution of imprinted genes to neurodevelopmental and neuropsychiatric disorders. Transl Psychiatry. 2022;12(1):210. 35597773 10.1038/s41398-022-01972-4 PMC9124202

[61] Koetsier J, Eijssen LMT, Schurgers LJ, Curfs LMG, Reutelingsperger CP, Bahram Sangani N. Integrative analysis of 115 transcriptomic studies decodes the molecular landscape of neurodevelopmental disorders. Commun Biol. 2025;8(1):914. 40500308 10.1038/s42003-025-08330-2 PMC12159135

[62] Prasasya R, Grotheer KV, Siracusa LD, Bartolomei MS. Temple syndrome and Kagami-Ogata syndrome: clinical presentations, genotypes, models and mechanisms. Hum Mol Genet. 2020;29(R1):R107–16. 32592473 10.1093/hmg/ddaa133 PMC8325017

[63] Stelzer Y, Sagi I, Yanuka O, Eiges R, Benvenisty N. The noncoding RNA IPW regulates the imprinted DLK1-DIO3 locus in an induced pluripotent stem cell model of Prader-Willi syndrome. Nat Genet. 2014;46(6):551–7. 24816254 10.1038/ng.2968

[64] Wawrzik M, Spiess AN, Herrmann R, Buiting K, Horsthemke B. Expression of SNURF-SNRPN upstream transcripts and epigenetic regulatory genes during human spermatogenesis. Eur J Hum Genet. 2009;17(11):1463–70. 19471314 10.1038/ejhg.2009.83 PMC2986690

[65] Jiang J, Wang X, Lu J. PWRN1 Suppressed Cancer Cell Proliferation and Migration in Glioblastoma by Inversely Regulating hsa-miR-21-5p. Cancer Manag Res. 2020;12:5313–22. 32753949 10.2147/CMAR.S250166 PMC7342408

[66] Shi J, Fu Q, Yang P, Yi Z, Liu S, Wang K. Long noncoding RNA PWRN1 is lowly expressed in osteosarcoma and modulates cancer proliferation and migration by targeting hsa-miR-214-5p. IUBMB Life. 2020;72(11):2444–53. 32870579 10.1002/iub.2370

[67] Lamar KJ, Carvill GL. Chromatin Remodeling Proteins in Epilepsy: Lessons From CHD2-Associated Epilepsy. Front Mol Neurosci. 2018;11:208. 29962935 10.3389/fnmol.2018.00208 PMC6013553

[68] Bergo A, Strollo M, Gai M, Barbiero I, Stefanelli G, Sertic S, et al. Methyl-CpG binding protein 2 (MeCP2) localizes at the centrosome and is required for proper mitotic spindle organization. J Biol Chem. 2015;290(6):3223–37. 25527496 10.1074/jbc.M114.608125 PMC4318997

[69] Bocchi R, Egervari K, Carol-Perdiguer L, Viale B, Quairiaux C, De Roo M, et al. Perturbed Wnt signaling leads to neuronal migration delay, altered interhemispheric connections and impaired social behavior. Nat Commun. 2017;8(1):1158. 29079819 10.1038/s41467-017-01046-w PMC5660087

[70] Long KR, Huttner WB. How the extracellular matrix shapes neural development. Open Biol. 2019;9(1):180216. 30958121 10.1098/rsob.180216 PMC6367132

[71] Andrews WD, Barber M, Parnavelas JG. Slit-Robo interactions during cortical development. J Anat. 2007;211(2):188–98. 17553100 10.1111/j.1469-7580.2007.00750.x PMC2375773

[72] Hatanaka Y, Murakami F. In vitro analysis of the origin, migratory behavior, and maturation of cortical pyramidal cells. J Comp Neurol. 2002;454(1):1–14. 12410614 10.1002/cne.10421

[73] Yildirim M, Delepine C, Feldman D, Pham VA, Chou S, Ip J et al. Label-free three-photon imaging of intact human cerebral organoids for tracking early events in brain development and deficits in Rett syndrome. Elife. 2022;11:e78079. 10.7554/eLife.78079 PMC9337854 35904330

[74] Kelava I, Lancaster MA. Dishing out mini-brains: Current progress and future prospects in brain organoid research. Dev Biol. 2016;420(2):199–209. 27402594 10.1016/j.ydbio.2016.06.037 PMC5161139

[75] Gafson AR, Barthélemy NR, Bomont P, Carare RO, Durham HD, Julien JP, et al. Neurofilaments: neurobiological foundations for biomarker applications. Brain. 2020;143(7):1975–98. 32408345 10.1093/brain/awaa098 PMC7363489

[76] Brás J, Gibbons E, Guerreiro R. Genetics of synucleins in neurodegenerative diseases. Acta Neuropathol. 2021;141(4):471–90. 32740728 10.1007/s00401-020-02202-1

[77] Puddifoot CA, Wu M, Sung RJ, Joiner WJ. Ly6h regulates trafficking of alpha7 nicotinic acetylcholine receptors and nicotine-induced potentiation of glutamatergic signaling. J Neurosci. 2015;35(8):3420–30. 25716842 10.1523/JNEUROSCI.3630-14.2015 PMC4339353

[78] Luo Y, Liao S, Yu J. Netrin-1 in Post-stroke neuroprotection: beyond axon guidance cue. Curr Neuropharmacol. 2022;20(10):1879–87. 35236266 10.2174/1570159X20666220302150723 PMC9886807

[79] Quinn JP, Kandigian SE, Trombetta BA, Arnold SE, Carlyle BC. VGF as a biomarker and therapeutic target in neurodegenerative and psychiatric diseases. Brain Commun. 2021;3(4):fcab261. 34778762 10.1093/braincomms/fcab261 PMC8578498

[80] Tang X, Jaenisch R, Sur M. The role of GABAergic signalling in neurodevelopmental disorders. Nat Rev Neurosci. 2021;22(5):290–307. 33772226 10.1038/s41583-021-00443-x PMC9001156

[81] Ure K, Lu H, Wang W, Ito-Ishida A, Wu Z, He LJ et al. Restoration of Mecp2 expression in GABAergic neurons is sufficient to rescue multiple disease features in a mouse model of Rett syndrome. Elife. 2016;5:e14198. 10.7554/eLife.14198 PMC4946897 27328321

[82] Yoo Y, Jung J, Lee YN, Lee Y, Cho H, Na E, et al. GABBR2 mutations determine phenotype in rett syndrome and epileptic encephalopathy. Ann Neurol. 2017;82(3):466–78. 28856709 10.1002/ana.25032

[83] Chao HT, Chen H, Samaco RC, Xue M, Chahrour M, Yoo J, et al. Dysfunction in GABA signalling mediates autism-like stereotypies and Rett syndrome phenotypes. Nature. 2010;468(7321):263–9. 21068835 10.1038/nature09582 PMC3057962

[84] Luján R, Shigemoto R, López-Bendito G. Glutamate and GABA receptor signalling in the developing brain. Neuroscience. 2005;130(3):567–80. 15590141 10.1016/j.neuroscience.2004.09.042

[85] Chiefari E, Foti DP, Sgarra R, Pegoraro S, Arcidiacono B, Brunetti FS, et al. Transcriptional regulation of glucose metabolism: the emerging role of the HMGA1 chromatin factor. Front Endocrinol (Lausanne). 2018;9:357. 30034366 10.3389/fendo.2018.00357 PMC6043803

[86] Gandhi S, Hutchins EJ, Maruszko K, Park JH, Thomson M, Bronner ME. Bimodal function of chromatin remodeler Hmga1 in neural crest induction and Wnt-dependent emigration. Elife. 2020;9:e57779. 10.7554/eLife.57779 PMC7591248 32965216

[87] Rahman MR, Petralia MC, Ciurleo R, Bramanti A, Fagone P, Shahjaman M et al. Comprehensive Analysis of RNA-Seq Gene Expression Profiling of Brain Transcriptomes Reveals Novel Genes, Regulators, and Pathways in Autism Spectrum Disorder. Brain Sci. 2020;10(10):747. 10.3390/brainsci10100747 PMC7603078 33080834

[88] Yuan ZF, Mao SS, Shen J, Jiang LH, Xu L, Xu JL, et al. Insulin-like growth factor-1 down-regulates the phosphorylation of FXYD1 and rescues behavioral deficits in a mouse model of Rett syndrome. Front Neurosci. 2020;14:20. 32063830 10.3389/fnins.2020.00020 PMC7000522

[89] Kaufmann WE, Sprouse J, Rebowe N, Hanania T, Klamer D, Missling CU. ANAVEX^®^2–73 (blarcamesine), a Sigma-1 receptor agonist, ameliorates neurologic impairments in a mouse model of Rett syndrome. Pharmacol Biochem Behav. 2019;187:172796. 31704481 10.1016/j.pbb.2019.172796

[90] Park Y, Page N, Salamon I, Li D, Rasin MR. Making sense of mRNA landscapes: translation control in neurodevelopment. Wiley Interdiscip Rev RNA. 2022;13(1):e1674. 34137510 10.1002/wrna.1674

[91] Han ZA, Jeon HR, Kim SW, Park JY, Chung HJ. Clinical characteristics of children with rett syndrome. Ann Rehabil Med. 2012;36(3):334–9. 22837968 10.5535/arm.2012.36.3.334 PMC3400872

[92] Petibon C, Malik Ghulam M, Catala M, Abou Elela S. Regulation of ribosomal protein genes: an ordered anarchy. Wiley Interdiscip Rev RNA. 2021;12(3):e1632. 33038057 10.1002/wrna.1632 PMC8047918

[93] Young JI, Hong EP, Castle JC, Crespo-Barreto J, Bowman AB, Rose MF, et al. Regulation of RNA splicing by the methylation-dependent transcriptional repressor methyl-CpG binding protein 2. Proc Natl Acad Sci U S A. 2005;102(49):17551–8. 16251272 10.1073/pnas.0507856102 PMC1266160

[94] Osenberg S, Karten A, Sun J, Li J, Charkowick S, Felice CA, et al. Activity-dependent aberrations in gene expression and alternative splicing in a mouse model of Rett syndrome. Proc Natl Acad Sci U S A. 2018;115(23):E5363–72. 29769330 10.1073/pnas.1722546115 PMC6003366

