# Supplementary material for: Multi-omics characterization of developing forebrain organoids unravels the dynamic molecular events of Rett syndrome pathogenesis
Source: J Neurodev Disord. 2026 Apr 21;18:33. doi: 10.1186/s11689-026-09699-9 (PMC13227629; doi:10.1186/s11689-026-09699-9)
Supplement: Supplementary file 1 — Additional file 1. [file 11689_2026_9699_MOESM1_ESM.pdf]

Additional file 1

Multi-Omics Characterization of Developing Forebrain Organoids Unravels the Dynamic Molecular Events of Rett Syndrome Pathogenesis

Jarno Koetsier*, Nasim Bahram Sangani*, Ana Rita Gomes, Maria Margarida Diogo, Tiago G. Fernandes, Freek G. Bouwman, Edwin C. M. Mariman, Mehrnaz Ghazvini, Leon J. Schurgers, Joost Gribnau, Leopold M.G. Curfs, Chris P. Reutelingsperger, Lars M.T. Eijssen

*These authors contributed equally: Jarno Koetsier, Nasim Bahram Sangani

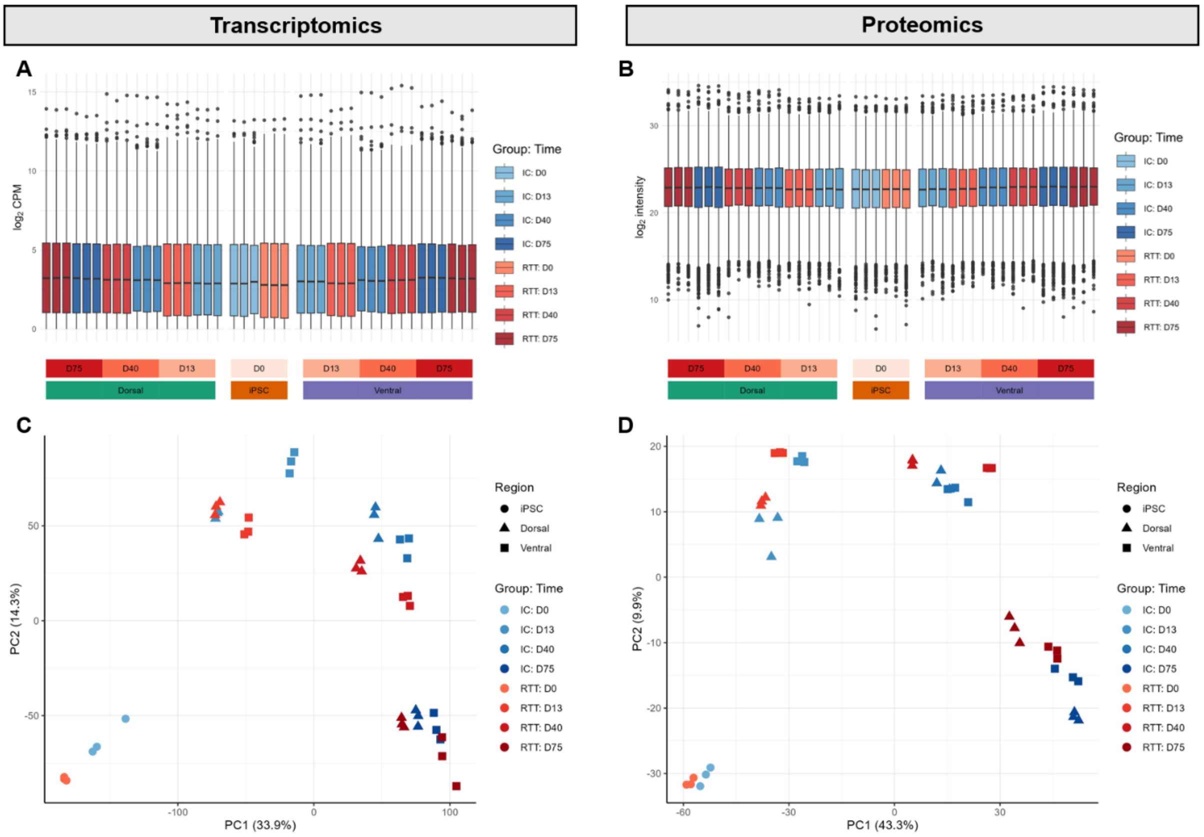

Figure S1. Quality control plots of the normalized transcriptomics and proteomics data.
(A) Boxplots of the distribution of the normalized log_2 counts per million (CPM) values for the different samples (transcriptomics).
(B) Boxplots of the distribution of the normalized log_2 intensity values for the different samples (proteomics).
(C) Principal Component Analysis (PCA) score plot of the transcriptomics data.
(D) PCA score plot of the proteomics data. The PCA plots in panels C and D both show grouping of samples by their experimental groups without the presence of any outliers.

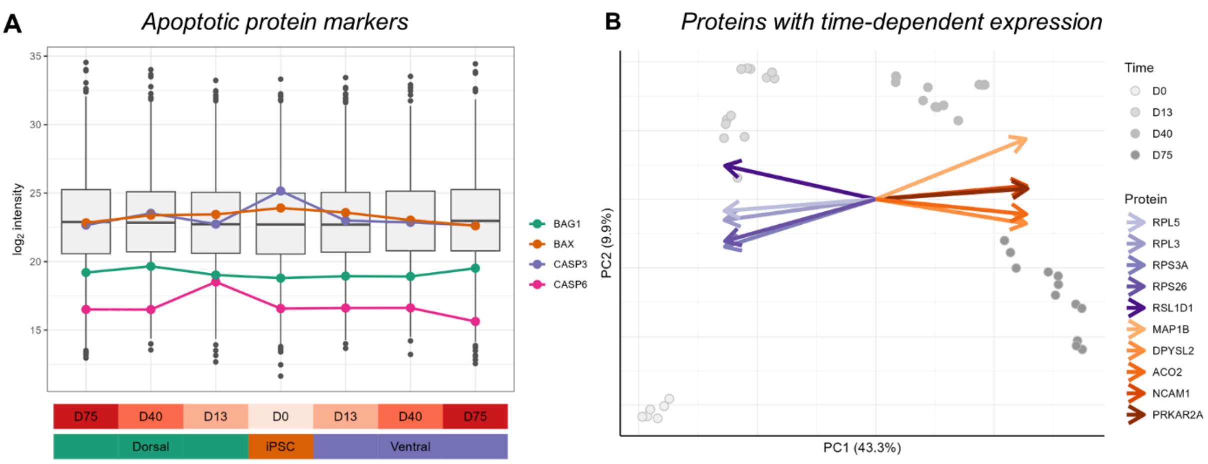

Figure S2. Quality control of the experimental model (proteomics).
(A) Mean expression (log_2 intensity) of pro-apoptotic (*i.e.,* BAX, CASP3, and CASP9) and anti-apoptotic (*i.e.,* BAG1) markers over time in IC samples. The grey-colored boxplots on the background indicate the distribution of the expression levels of all proteins that passed QC.
(B) Biplot of the PCA scores and the loadings of the five proteins with highest positive (orange arrows) and negative (purple arrow) PC1 loadings. The arrows indicate the proteins' contributions to the principal components.

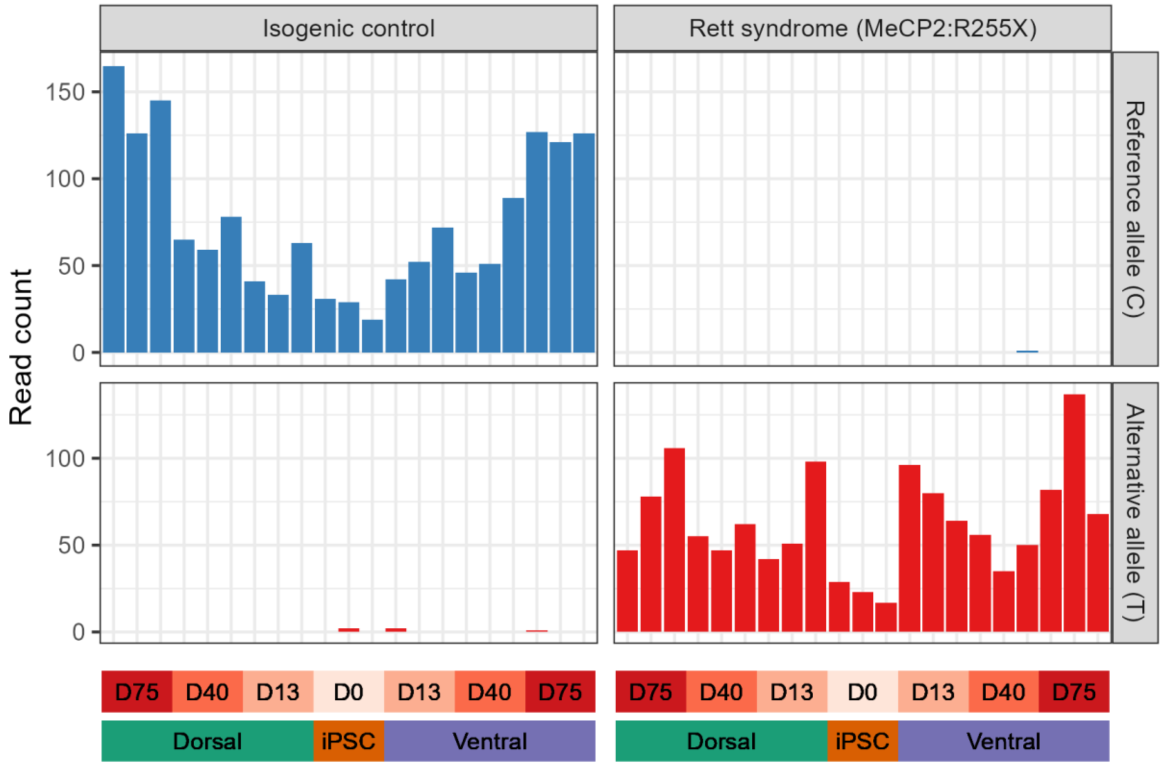

Figure S3. Allele-specific expression of the *MECP2* gene.
The number of reads carrying the reference (C) and alternative (T) allele (rs61749721) is shown for the Rett syndrome and isogenic control samples. The expression of the reference allele is limited to the isogenic control samples, while the expression of the alternative allele is specific to the Rett syndrome samples.

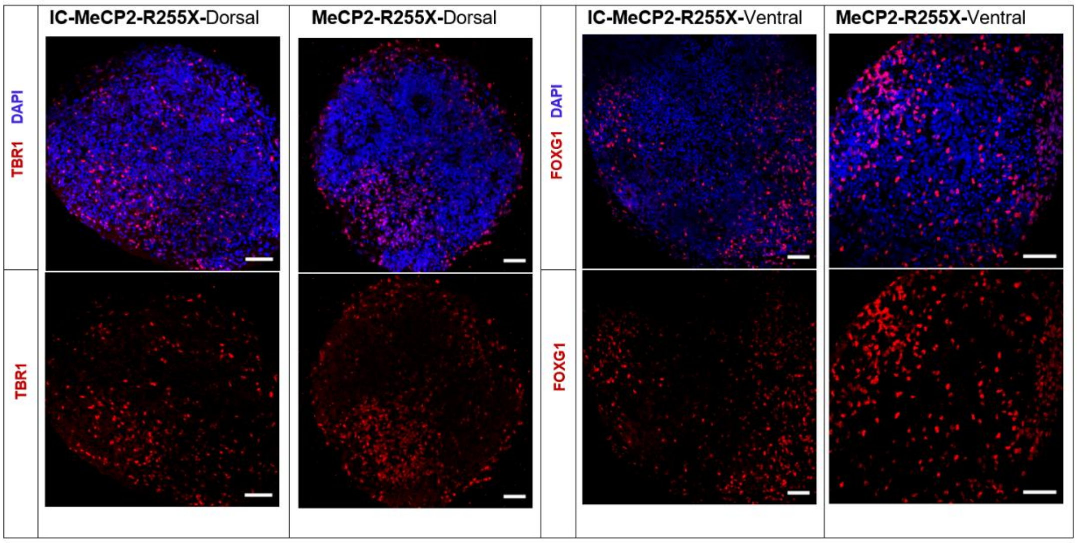

Figure S4. Immunofluorescence characterization of the forebrain organoids.
Representative images of immunofluorescence characterization of the ventral and dorsal forebrain organoids at day 41. The dorsal organoid sections were immunostained against the deep cortical layer marker TBR1. TBR1 is a transcription factor regulating cortical development and is mainly expressed in early-born postmitotic neurons. The ventral organoid sections were immunostained against the FOXG1, a generic forebrain marker that is highly expressed in ventral region. Furthermore, DAPI was used for nuclear staining. The scale bars represent 50 µm. Organoid sections were prepared and immunostained as previously described[1,2]. These studies also include a more elaborate characterization of MeCP2:R255X and IC-MeCP2:R255X ventral and dorsal forebrain organoids.

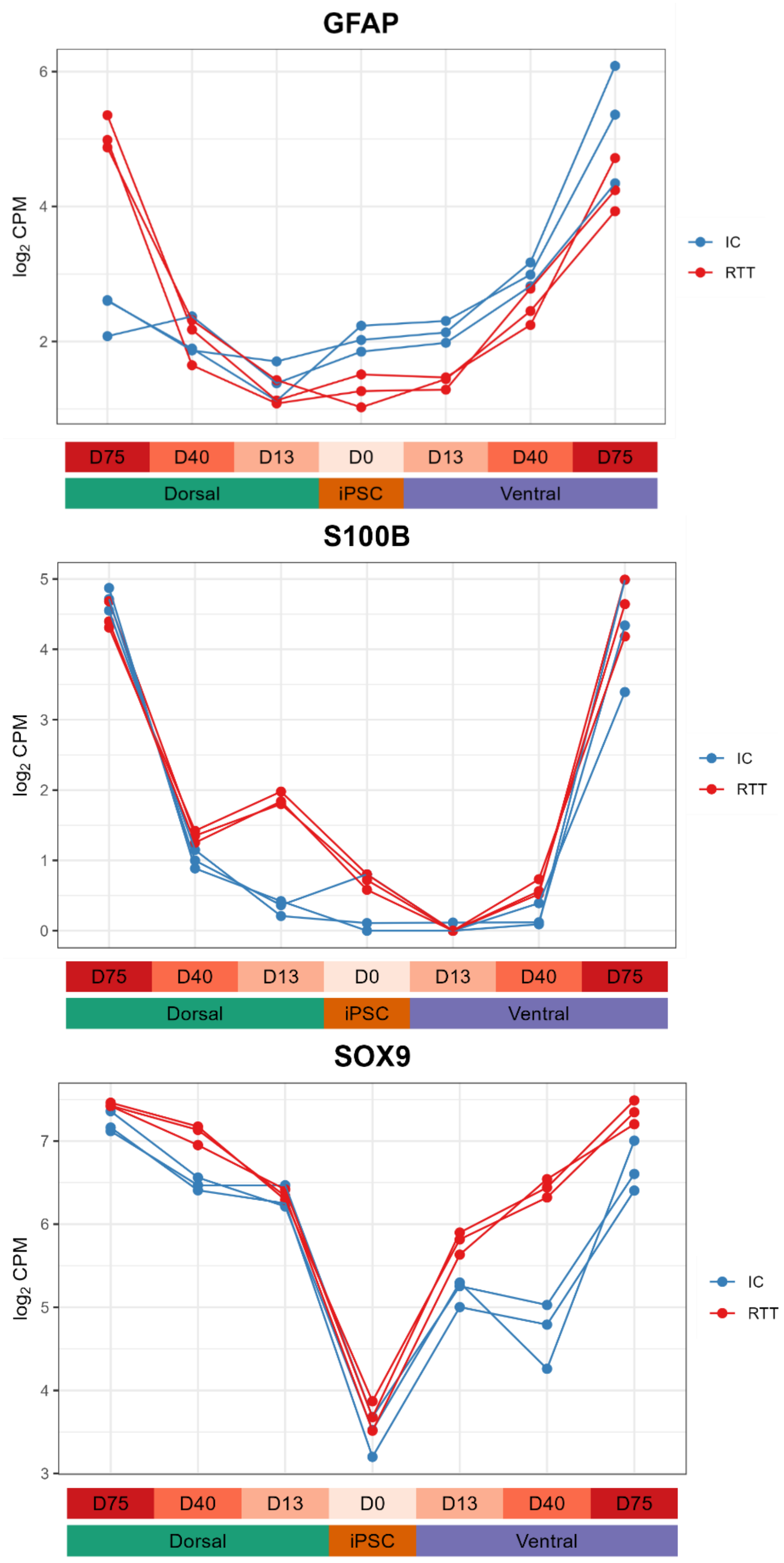

Figure S5. Spatiotemporal expression profile of the astrocyte markers *GFAP*, *S100B*, and *SOX9*.
The gene expression levels (*i.e.,* normalized log_2 CPM value) of the astrocyte markers *GFAP, S100B,* and *SOX9* is shown for RTT and IC ventral and dorsal forebrain organoids across the neurodevelopmental stages (days 0, 13, 40, and 75). The increasing expression of these markers over time suggests the presence of astrocytes at the later neurodevelopmental stages.

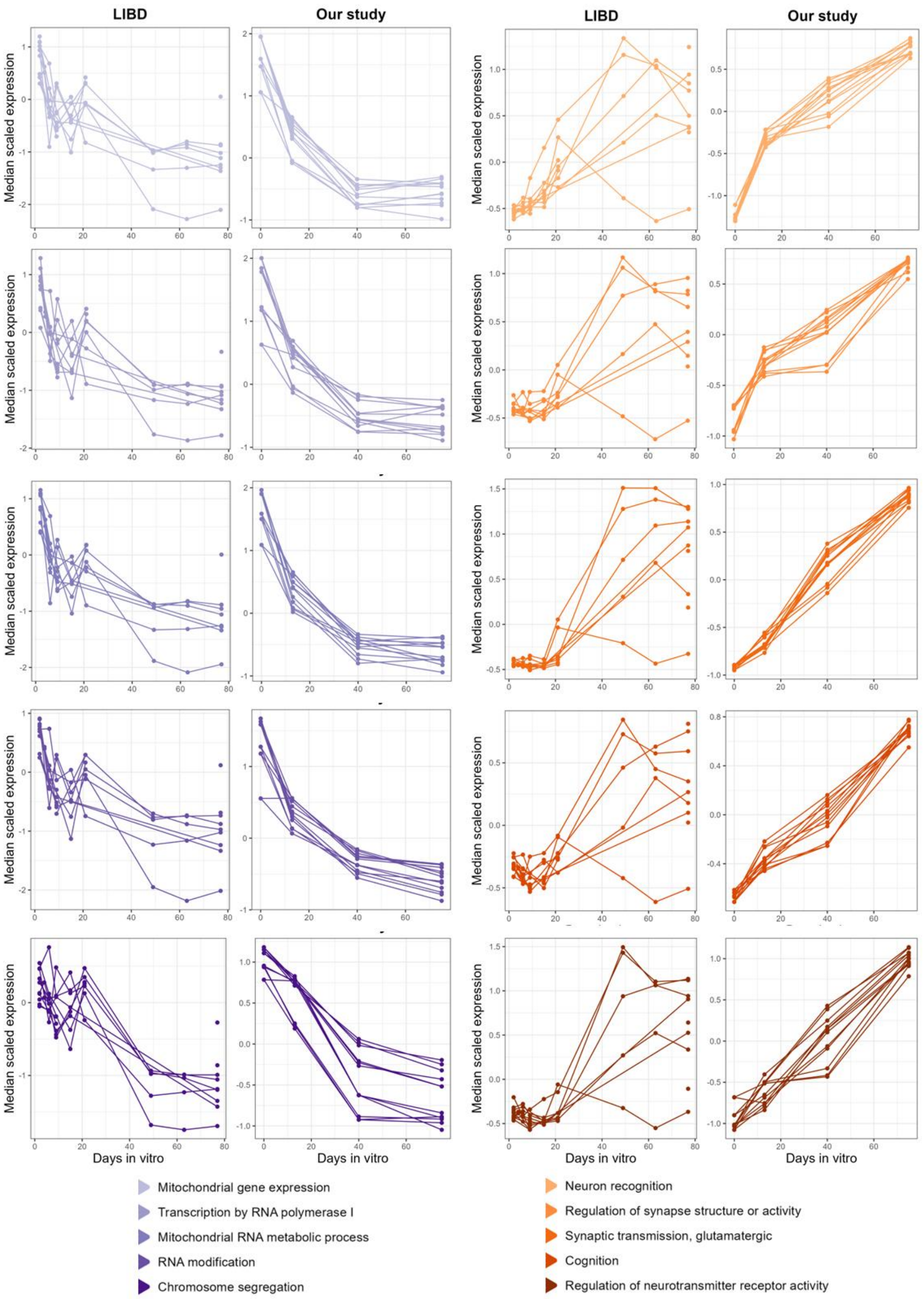

Figure S6. Validation of temporal expression profile.

The expression profile during neural differentiation is shown for the GO terms that were identified as having a decreasing (purple) and increasing (orange) expression over time (see Figure 4 of the main text). The expression profile of our study is compared to the LIBD Stem Cell Browser (https://stemcell.libd.org/scb/, control samples only). For each gene, the expression levels were mean-centered and unit variance scaled across all samples, after which the median value of each GO term was taken as the representative expression value for each sample. Each line represents a sample. There are 12 samples in our study (i.e., 3 IC-dorsal, 3 IC-ventral, 3 RTT-dorsal, 3 RTT-ventral samples) and 14 control samples from the LIBD Stem Cell Browser.

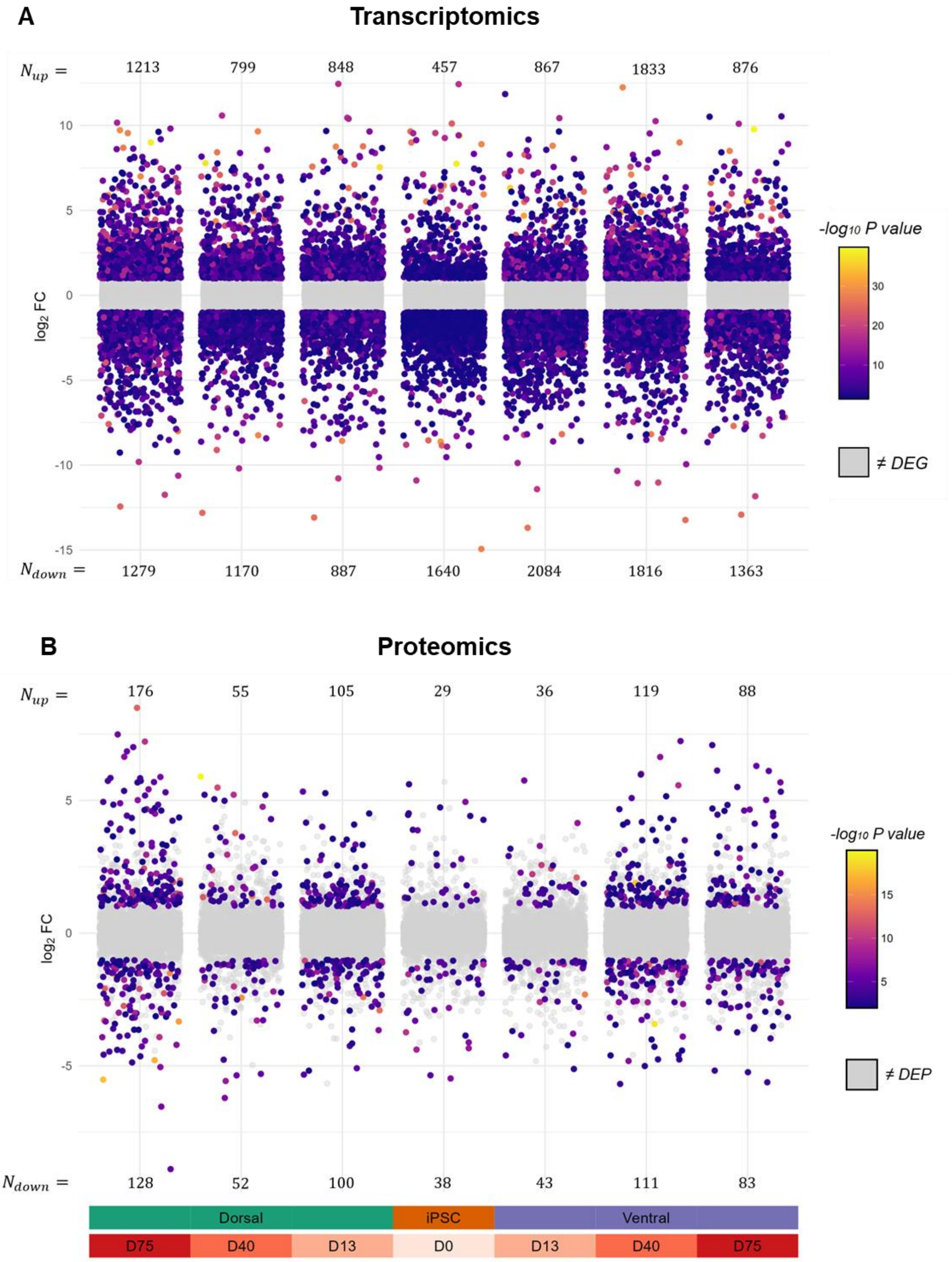

Figure S7. Differentially expressed genes (DEGs) and proteins (DEPs) in RTT versus IC.
(A) Scatter plot of the distribution of log_2FCs (vertical axis) and $-log_{10}$ P values (color gradient) at each time point and brain region for the transcriptomics data. The number of upregulated (N_{up}) and downregulated (N_{down}) DEGs at each time point and brain region are indicated at the top and bottom of the graph, respectively. DEGs were defined by an FDR-adjusted P value < 0.05 and $|log_2FC|>1$.
(B) Scatter plot of the distribution of log_2FCs (vertical axis) and $-log_{10}$ P values (color gradient) at each time point and brain region for the proteomics data. The number of upregulated (N_{up}) and downregulated (N_{down}) DEPs at each time point and brain region are indicated at the top and bottom of the graph, respectively. DEPs were defined by an FDR-adjusted P value < 0.05 and $|log_2FC|>1$.

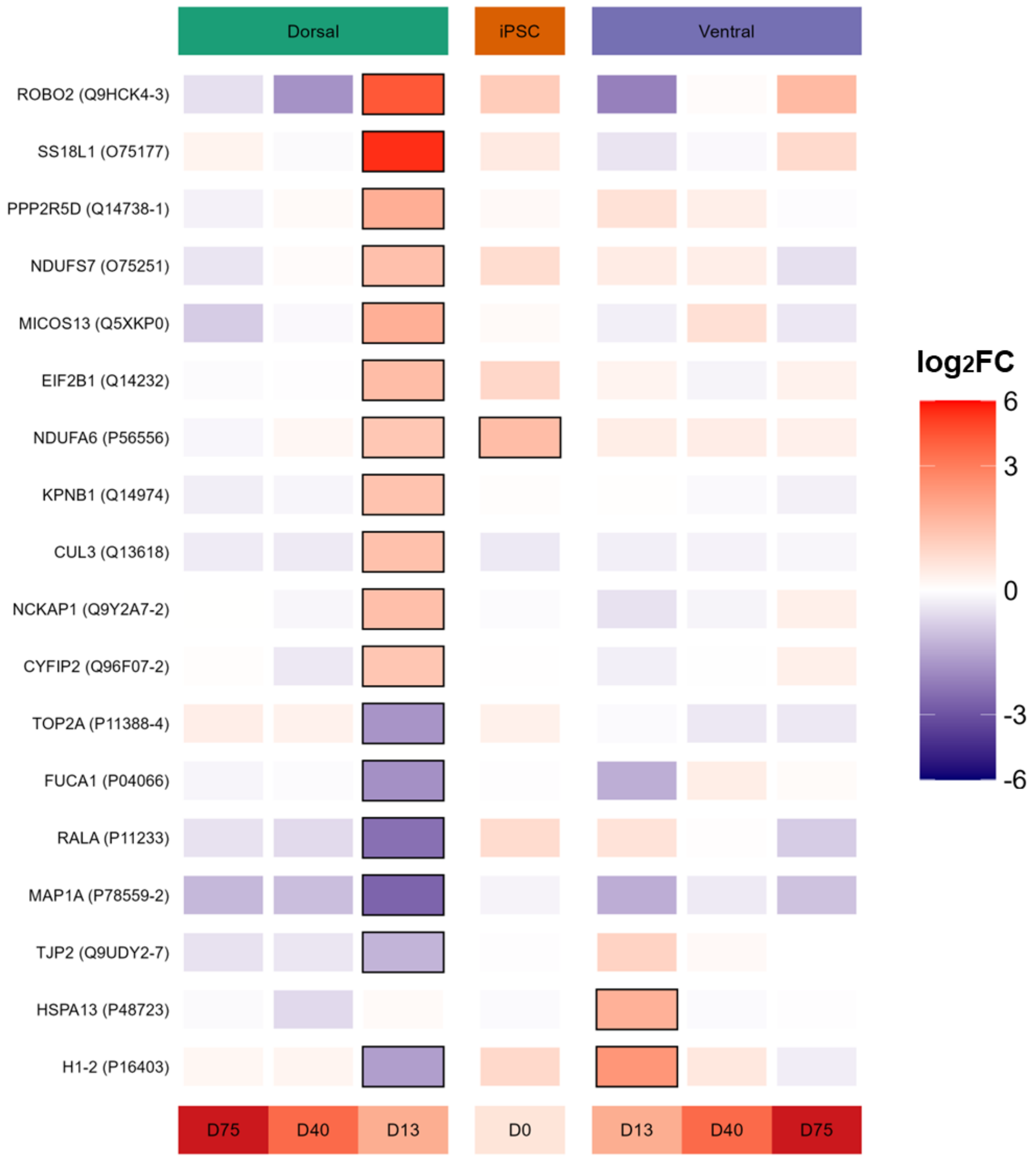

Figure S8. Differential protein expression profile of the neural progenitor cell (NPC)-specific protein markers.

These markers are specifically differentially expressed between RTT and IC at day 13 (*i.e.*, $|log_2FC| > 1$ and FDR-adjusted P value < 0.05), but not at the later developmental stages. The black border around the heatmap tiles indicates an FDR-adjusted P value < 0.05.

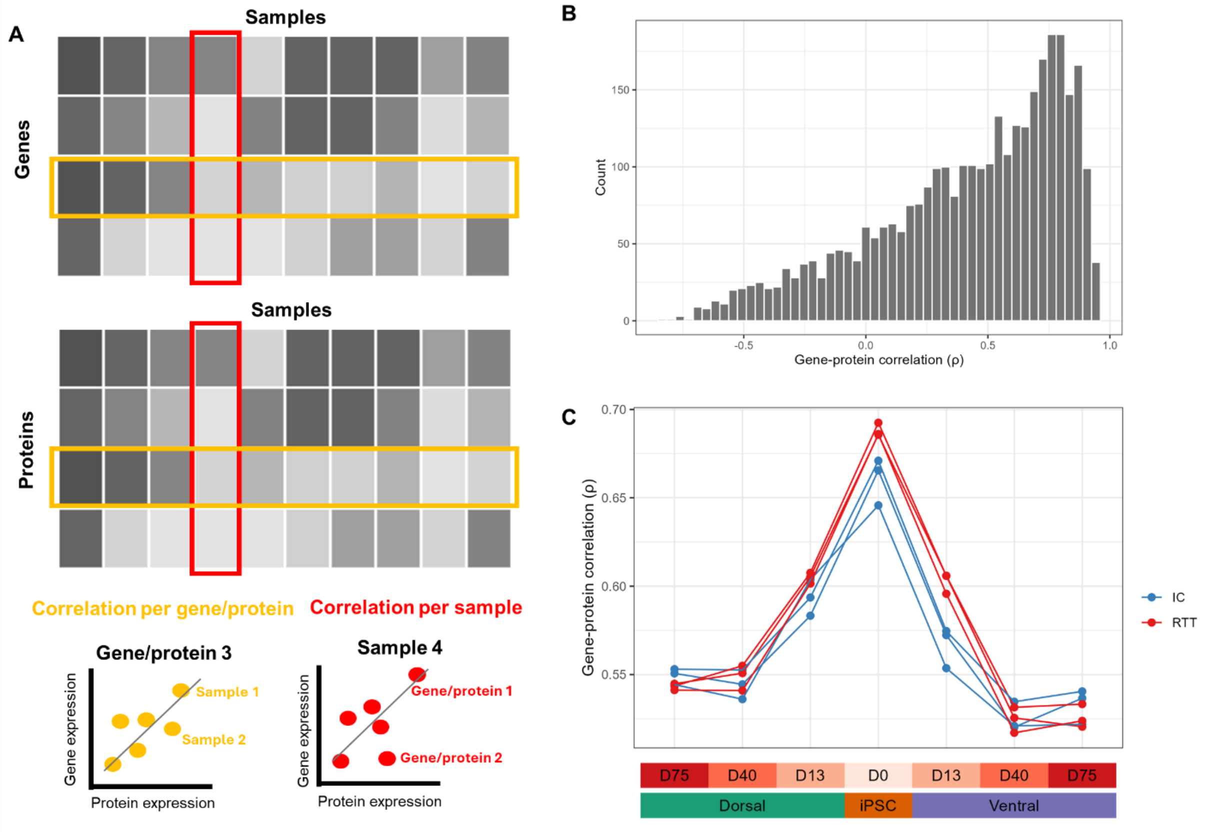

Figure S9. Gene-protein correlations across samples and genes.

(A) Schematic overview of how correlations were calculated per gene-protein pair across all samples (yellow) and per sample across all gene-protein pairs (red). The $\log_2$ FPKM values were used as the measure for gene expression, while $\log_2$ intensity values were used as the measure for protein expression. The Spearman correlation was used to quantify the relationship between gene and protein expression.

(B) Histogram of the distribution of gene-protein correlations per gene-protein pair.

(C) Gene-protein correlations per sample. Gene-protein correlations decrease during neural differentiation.

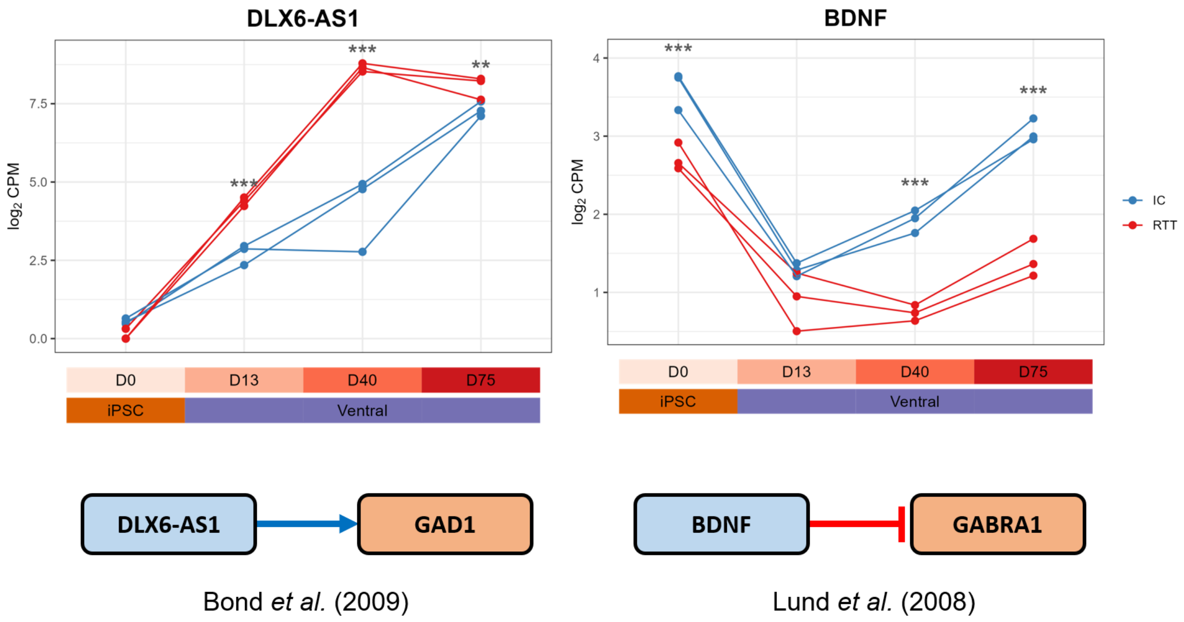

Figure S10. Temporal RNA expression profiles of DLX-AS1 and BDNF.
DLX-AS1 and BDNF which are known positive and negative transcriptional regulators of GABAergic signaling, respectively.[3,4]

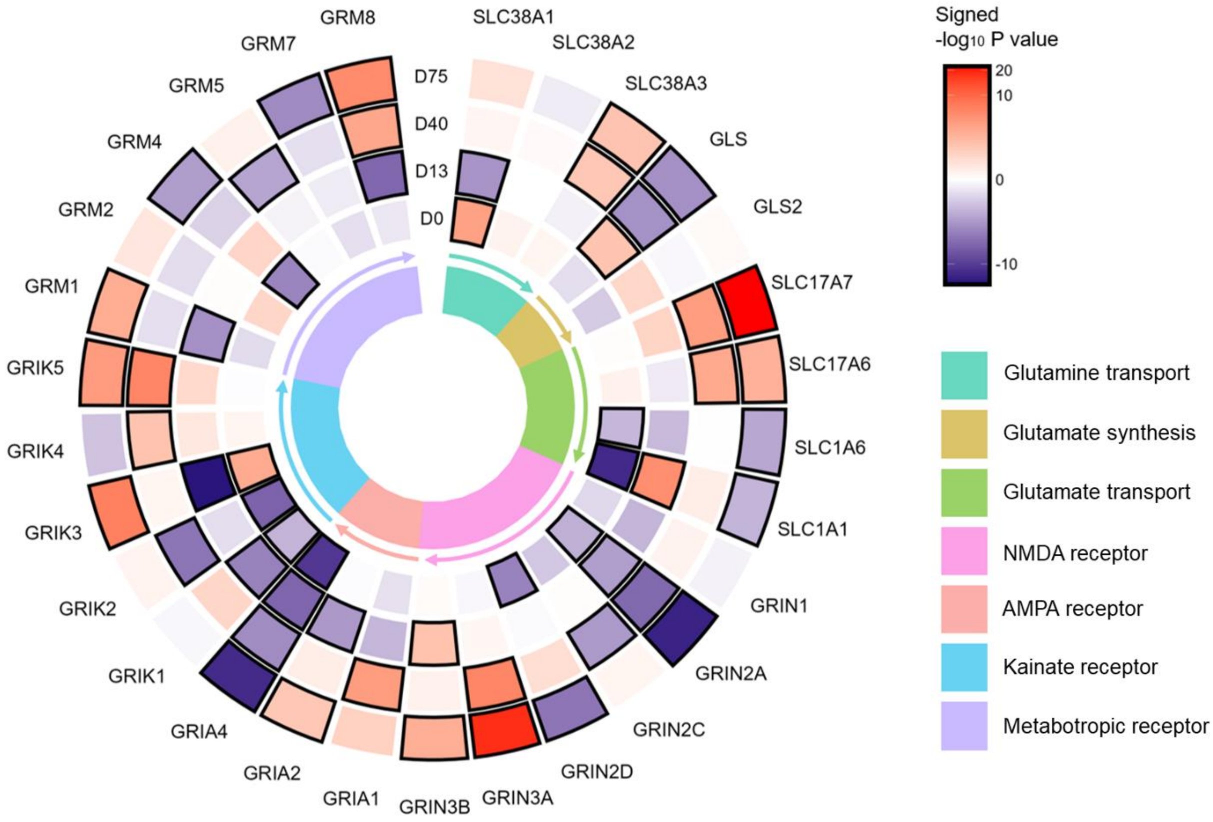

Figure S11. Circular heatmap of glutamatergic signaling-associated genes.
The heatmap is colored by the signed -$\log_{10}$ P value (*i.e.*, -$\log_{10}$ P value * sign $\log_2$FC) for the comparison RTT versus IC. The black border around the heatmap tiles indicates an FDR-adjusted P value < 0.05.

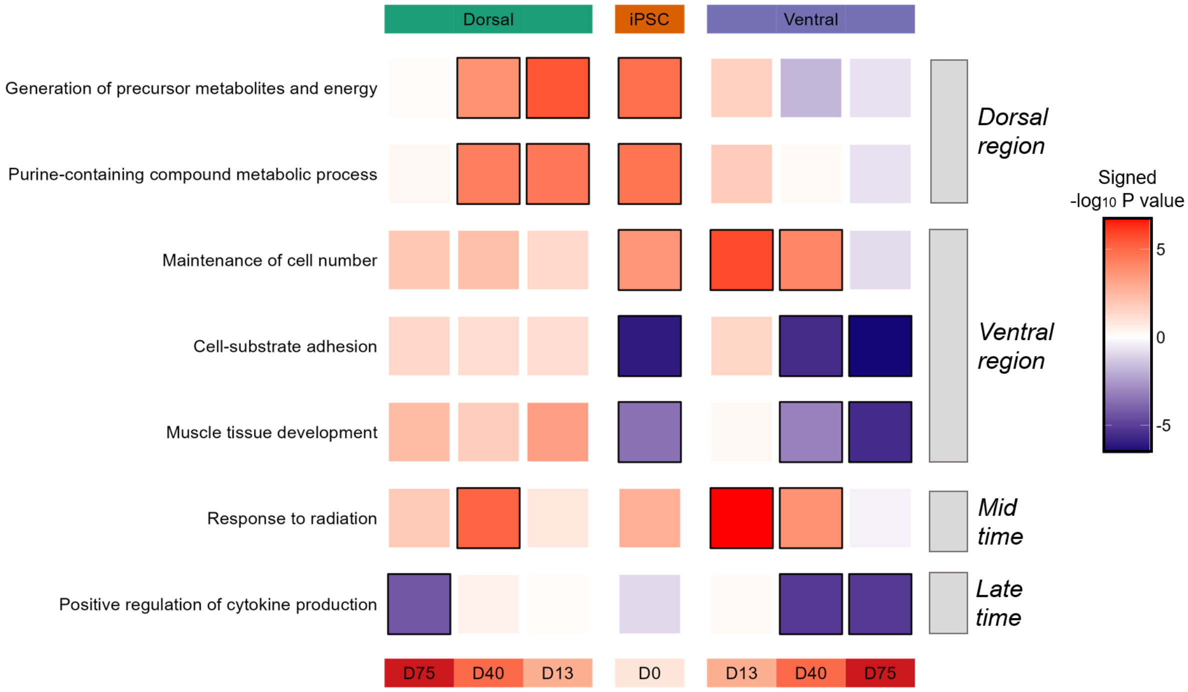

Figure S12. Time- and brain region-specific GO-BP terms.
The heatmap shows the signed -log$_{10}$ P value of the GO terms with a time- and or region-specific differential expression profile (Table S1). The sign of the signed -log$_{10}$ P value is determined by the enrichment score, where a negative and a positive score indicate an overrepresentation of the GO term's genes among the down- and upregulated genes, respectively. The black border around the heatmap tiles indicates an FDR-adjusted P value < 0.05.

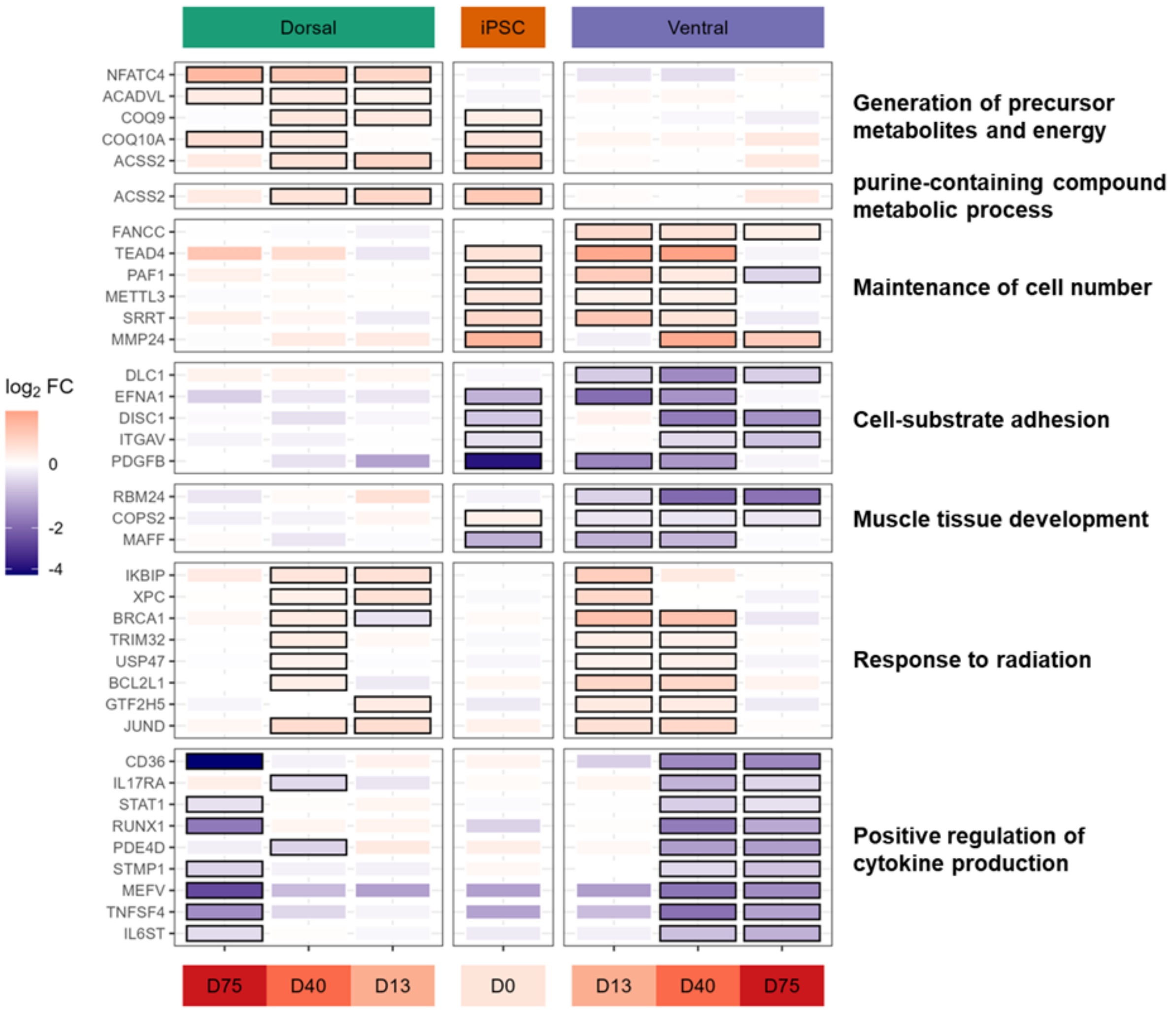

Figure S13. Time- and brain region-specific genes.
The heatmap shows the log_2FCs of the genes annotated to the time- and region-specific GO-BP terms from Figure S12 that exhibit the same time- and region-specific differential expression profile as their GO-BP term. The black border around the heatmap tiles indicates an FDR-adjusted P value < 0.05.

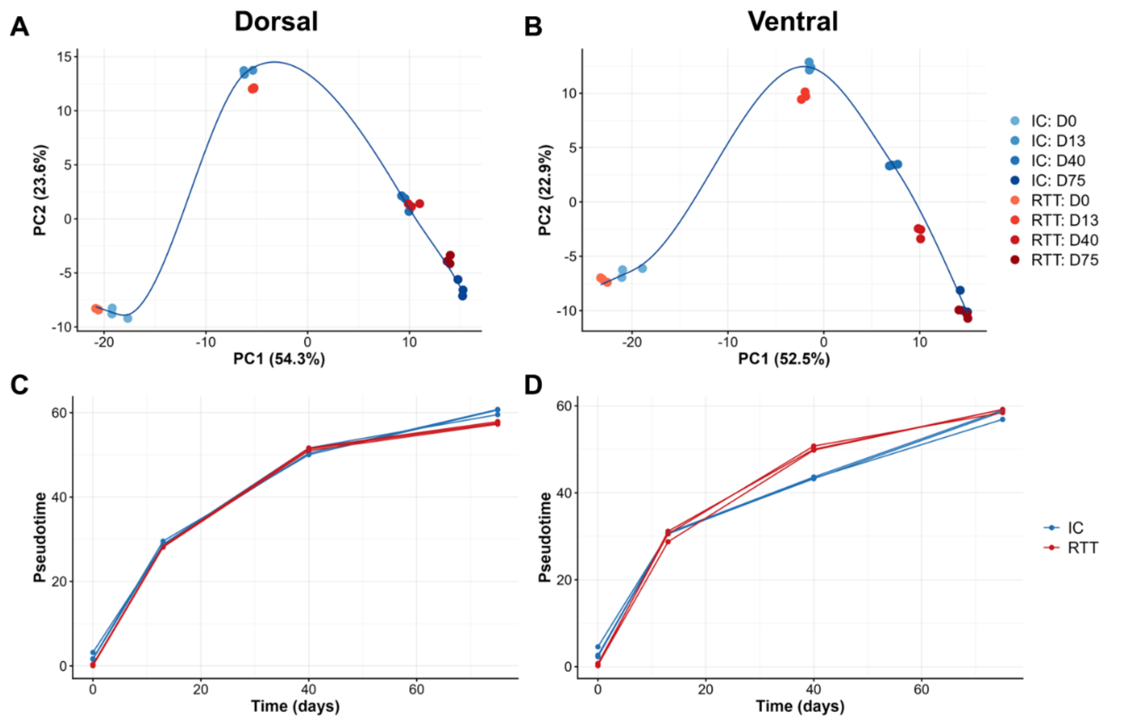

Figure S14. Trajectory inference analysis.

Pseudotime was estimated by performing PCA on genes associated with forebrain development (GO:0030900) using IC samples only. A smoothing spline was fitted through the IC samples to model the reference developmental trajectory (blue curve in panels A and B). RTT samples were subsequently projected into the same PCA space, and their pseudotime values were inferred by orthogonal projection onto the fitted spline. Pseudotime was defined as the distance along the spline (panels C and D). Trajectory inference was performed separately for dorsal and ventral forebrain organoids. A significant difference in pseudotime between RTT and IC samples, as estimated by Welch's two-sample t-test, was observed at day 40 in ventral organoids (P value = 4.4e-4) and at day 75 in dorsal organoids (P value = 9.4e-3). Although a more detailed characterization of developmental trajectories would require additional samples and time points, these findings suggest that some of the differences between RTT and IC forebrain organoids might be caused by a different developmental timing.

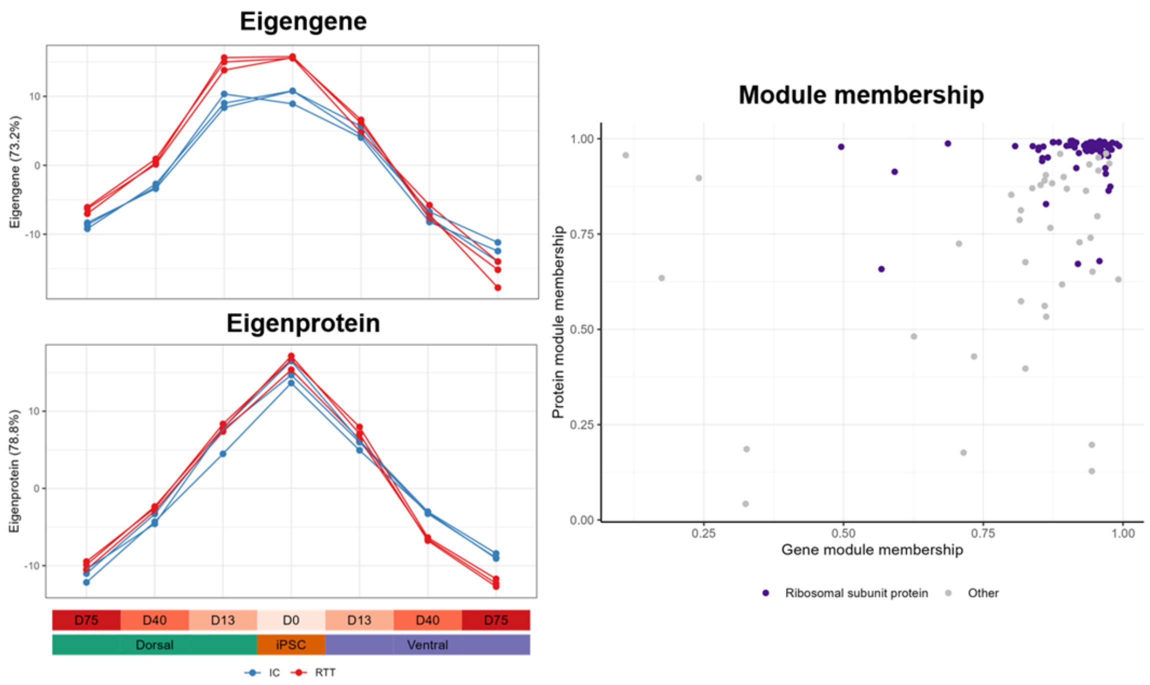

Figure S15. Spatiotemporal expression profile of the eigengene and eigenprotein of the *Cytoplasmic translation (GO:0002181)* term.
The eigengene and eigenprotein are the first principal component of the expression data of the genes and proteins in the cytoplasmic translation term, respectively. The Pearson correlation of the genes and proteins with the eigengene and eigenprotein (*i.e.,* module membership) is shown for the members of the cytoplasmic translation term. The large and small ribosomal subunit proteins (highlighted in purple, genenames.org) were found to have a high gene and protein module membership.

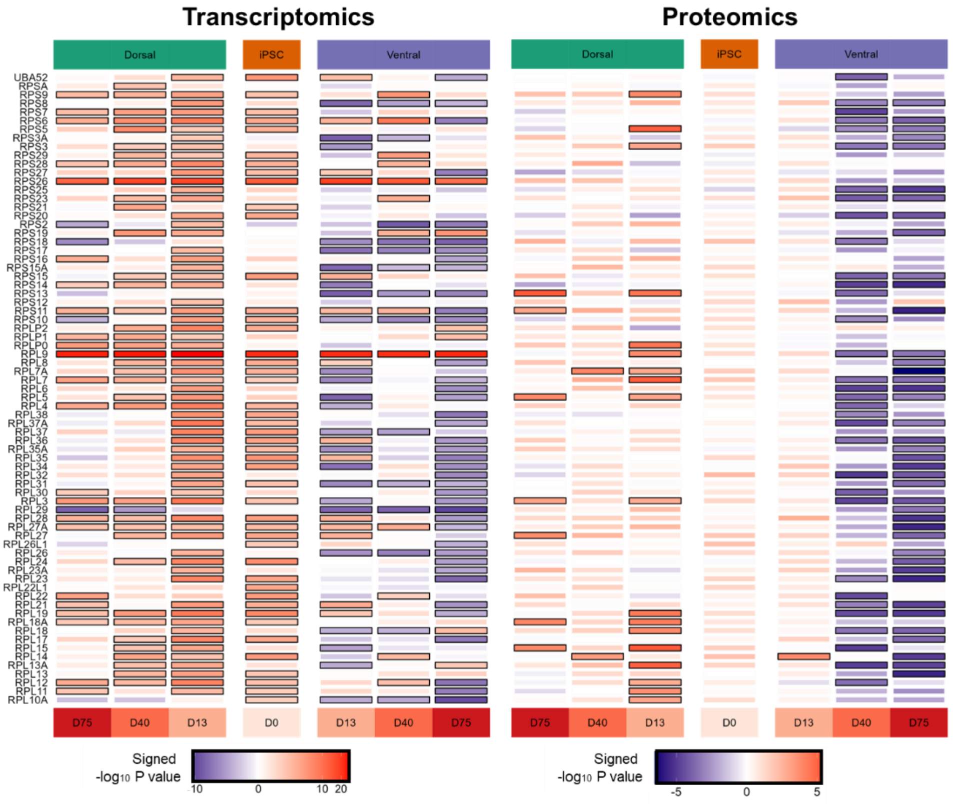

Figure S16. Heatmap of the signed -log$_{10}$ P value of the large and small ribosomal subunit protein.
The black border around the heatmap tiles indicates an FDR-adjusted P value < 0.05.

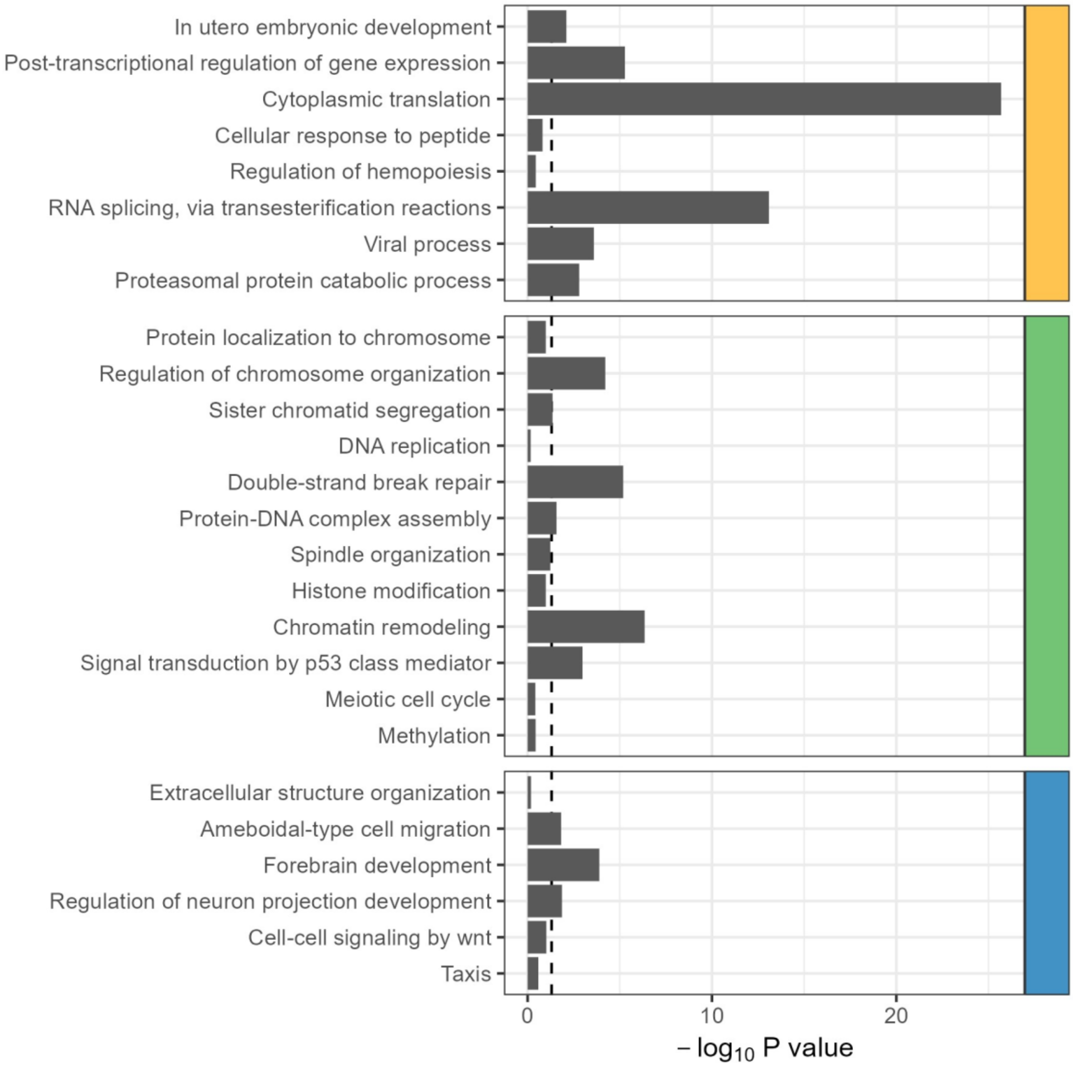

Figure S17. Statistical significance (*i.e.,* -$\log_{10}$ P value) of the identified 26 GO-BP for the overrepresentation analysis on the genes with differential isoform usage (DIU).

15 of the 26 GO terms are significantly enriched with the DIU genes, which is significantly more than expected by chance (permutation P value < 0.001).

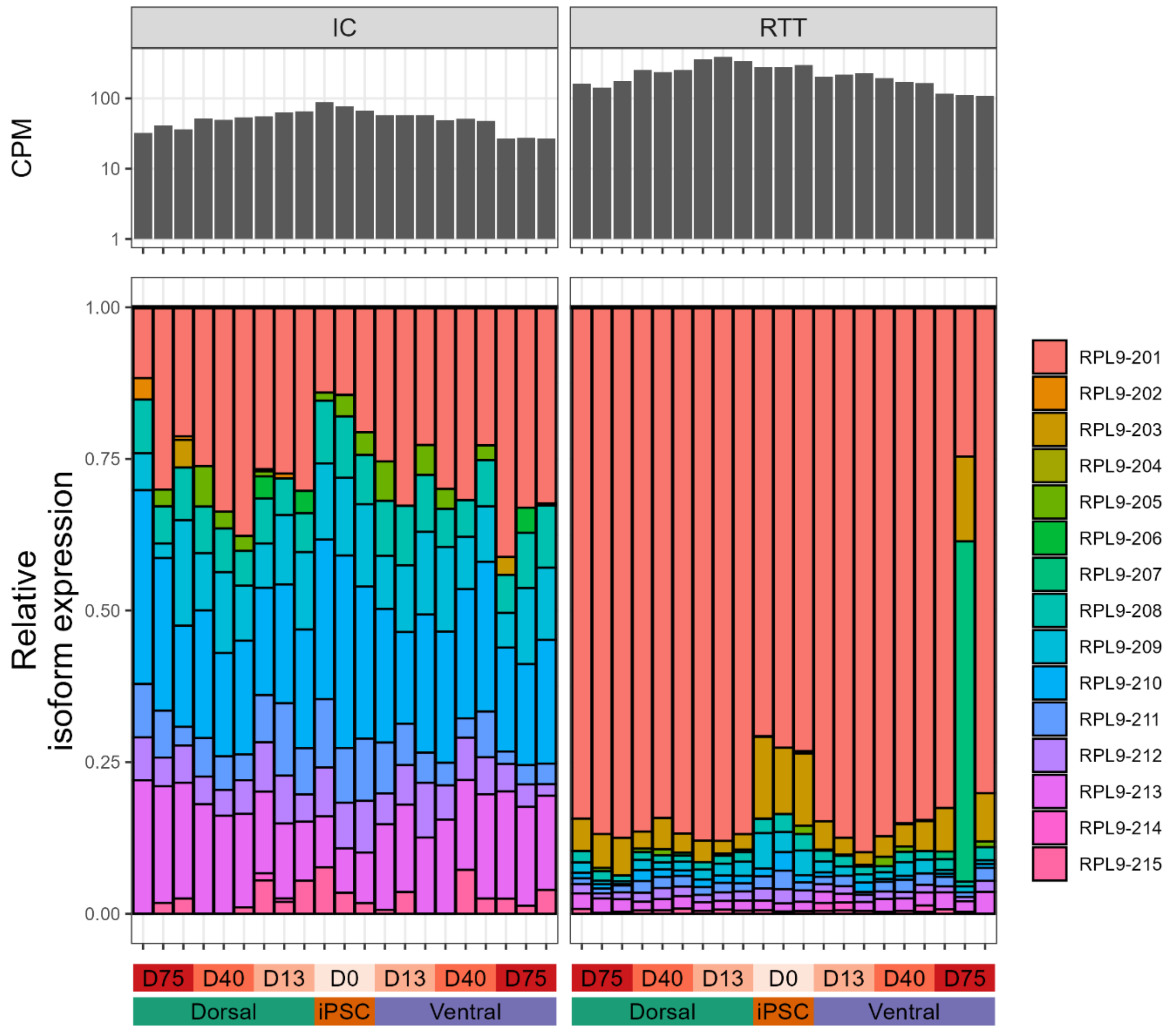

Figure S18. The total RPL9 expression (top panel) and the relative isoform abundances of the *RPL9* isoforms (bottom panel) in RTT and IC forebrain organoids.

The protein-coding RPL9 201 and -203 isoforms are predominantly responsible for the increased RPL9 expression in RTT.

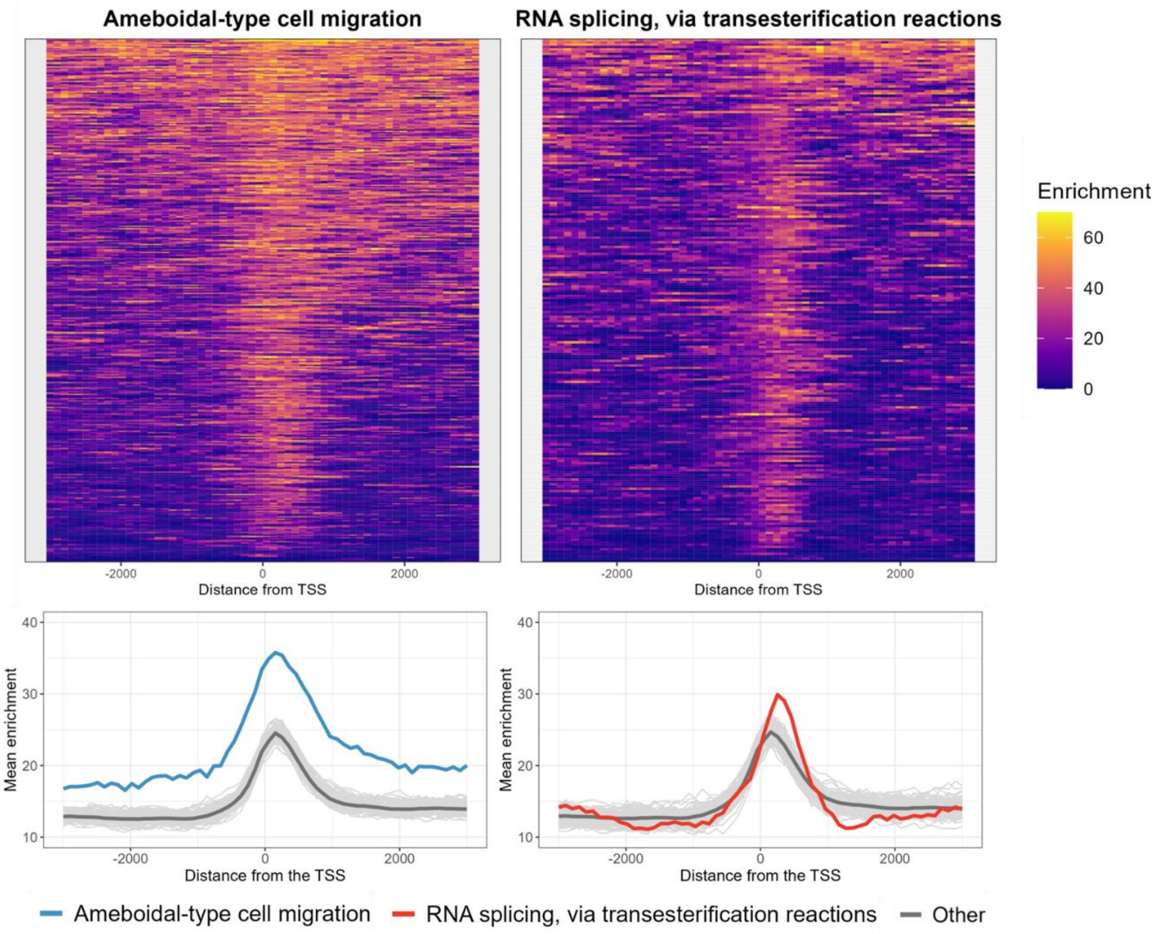

Figure S19. MeCP2 ChIP-seq enrichment.

The top panels show the MeCP2 enrichment of the genes annotated to the *Ameboidal-type cell migration (GO:0001667)* (n = 398) and *RNA splicing, via transesterification reaction* terms *(GO:0000375)* (n = 225). The mean MeCP2 enrichment of the genes annotated to these GO terms (blue and red, respectively) versus the enrichment of 100 random gene sets (grey) is visualized in the bottom right panel. For *Ameboidal-type cell migration*, the promoter regions are significantly enriched with MeCP2, while for *RNA splicing, via transesterification reaction*, the MeCP2 enrichment in the promoter region is not much different than for a random set of genes.

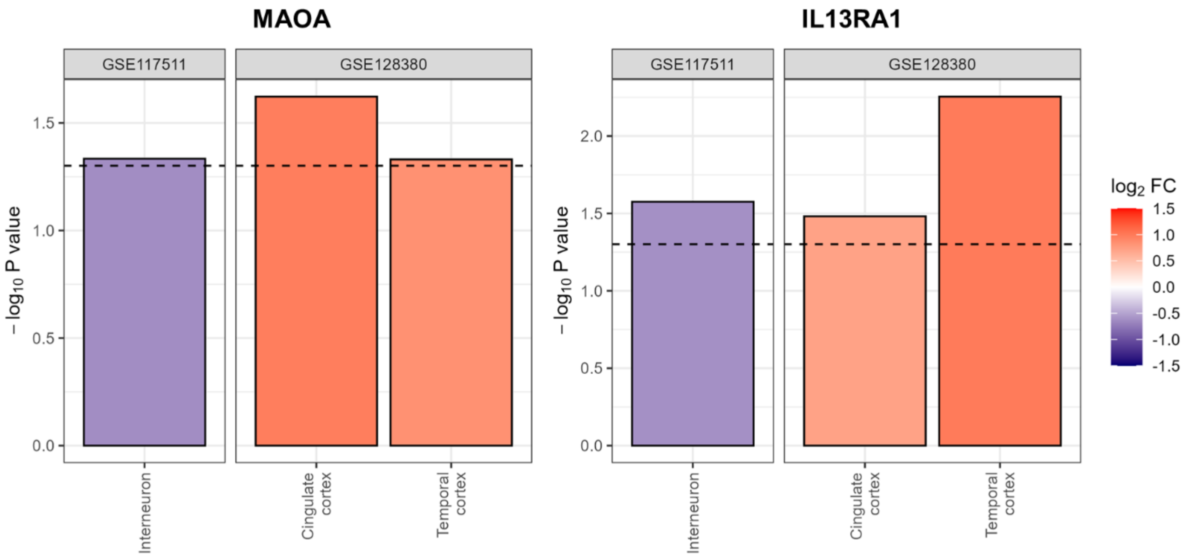

Figure S20. Differential expression profile of the X-linked genes MAOA and IL13RA1 across the male and post-mortem RNA-seq validation datasets.

MAOA and IL13RA are differentially expressed (*i.e.,* P value < 0.05) in all male (GSE117511) and post-mortem (GSE128380) validation comparisons. No other X-linked genes demonstrated differential expression across all validation comparisons. The dashed line indicates a P value threshold of 0.05.

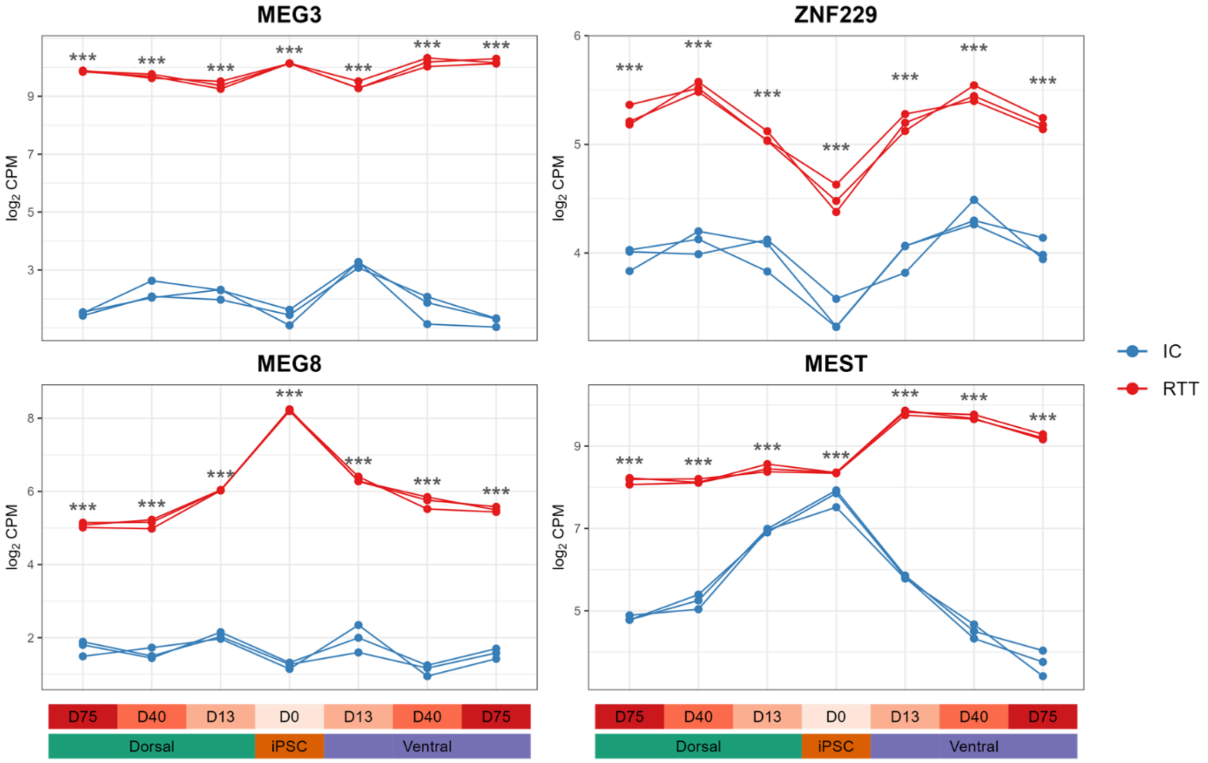

Figure S21. Spatiotemporal gene expression profile of four imprinted genes.
These four imprinted genes were differentially expressed (*i.e.,* FDR-adjusted P value < 0.05 and $|\log_2FC| > 1$) at minimally six of the seven time points and/or brain regions. * FDR-adjusted P value < 0.05, ** FDR-adjusted P value < 0.01, *** FDR-adjusted P value < 0.001.

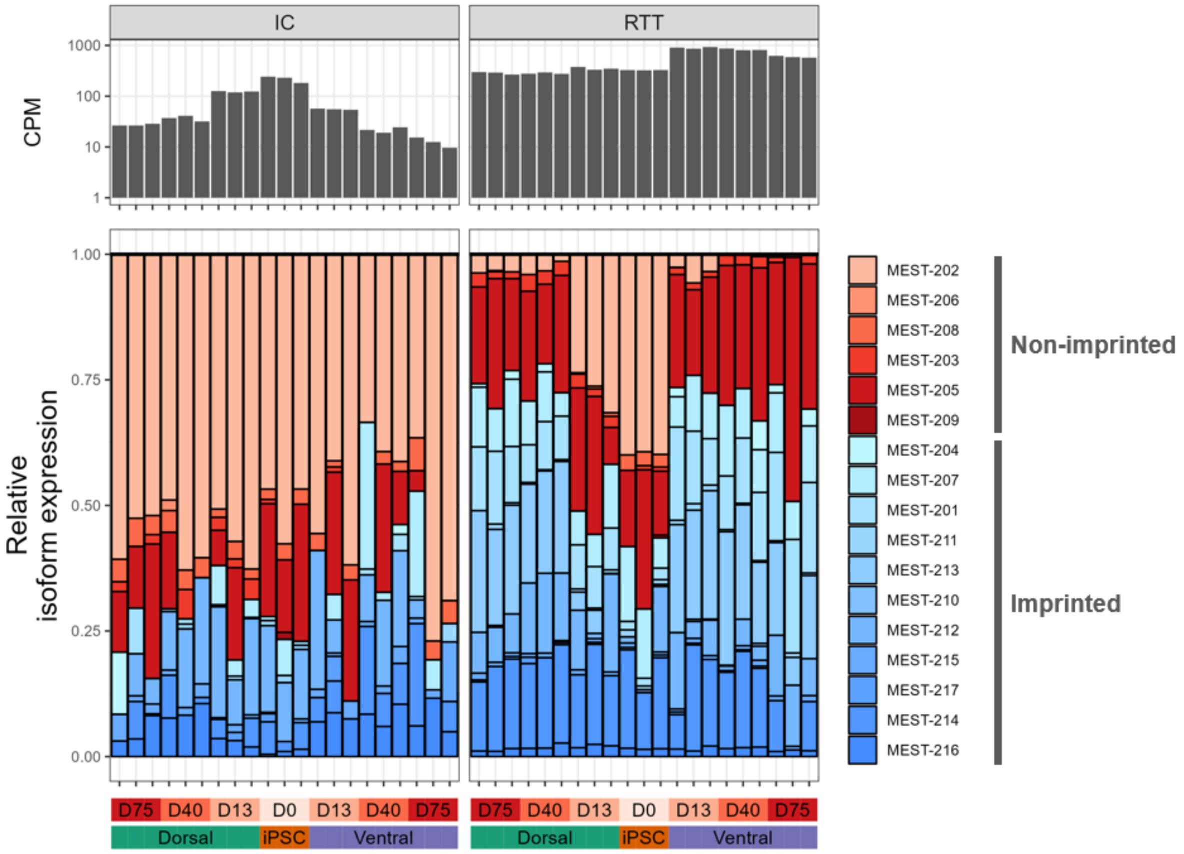

Figure S22. Relative isoform abundance of the *MEST* isoforms.
The isoforms are ordered based on their genomic location and colored based on whether or not they are transcribed from the imprinted or non-imprinted promoter.

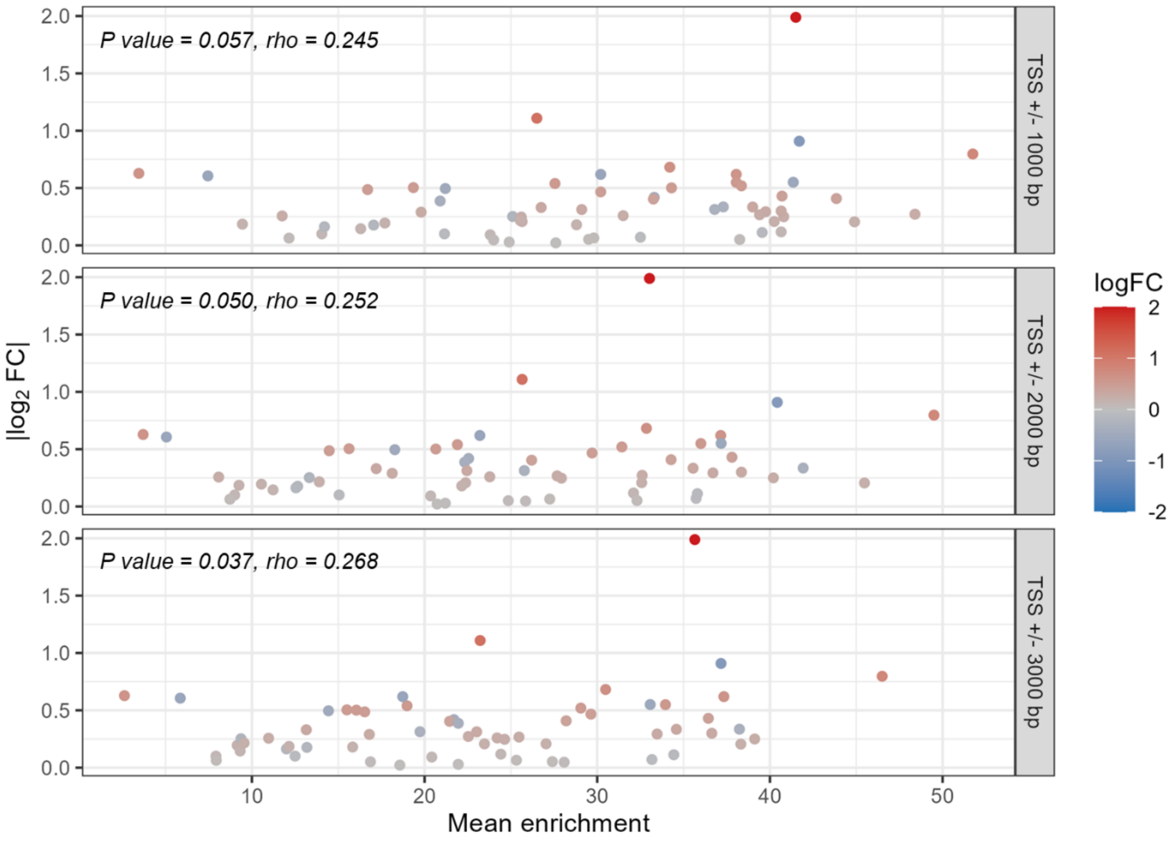

Figure S23. Association between MeCP2 enrichment around the transcription start site (TSS) and the absolute log_2FC in the olfactory neuroepithelia of MeCP2 knockout versus wildtype mice (dataset: GSE71126).

The mean MeCP2 enrichment was calculated for 1000 (top), 2000 (middle), and 3000 (bottom) base pairs from the TSS. The Spearman correlation was used to quantify the association between the absolute log_2FC and the mean MeCP2 enrichment.

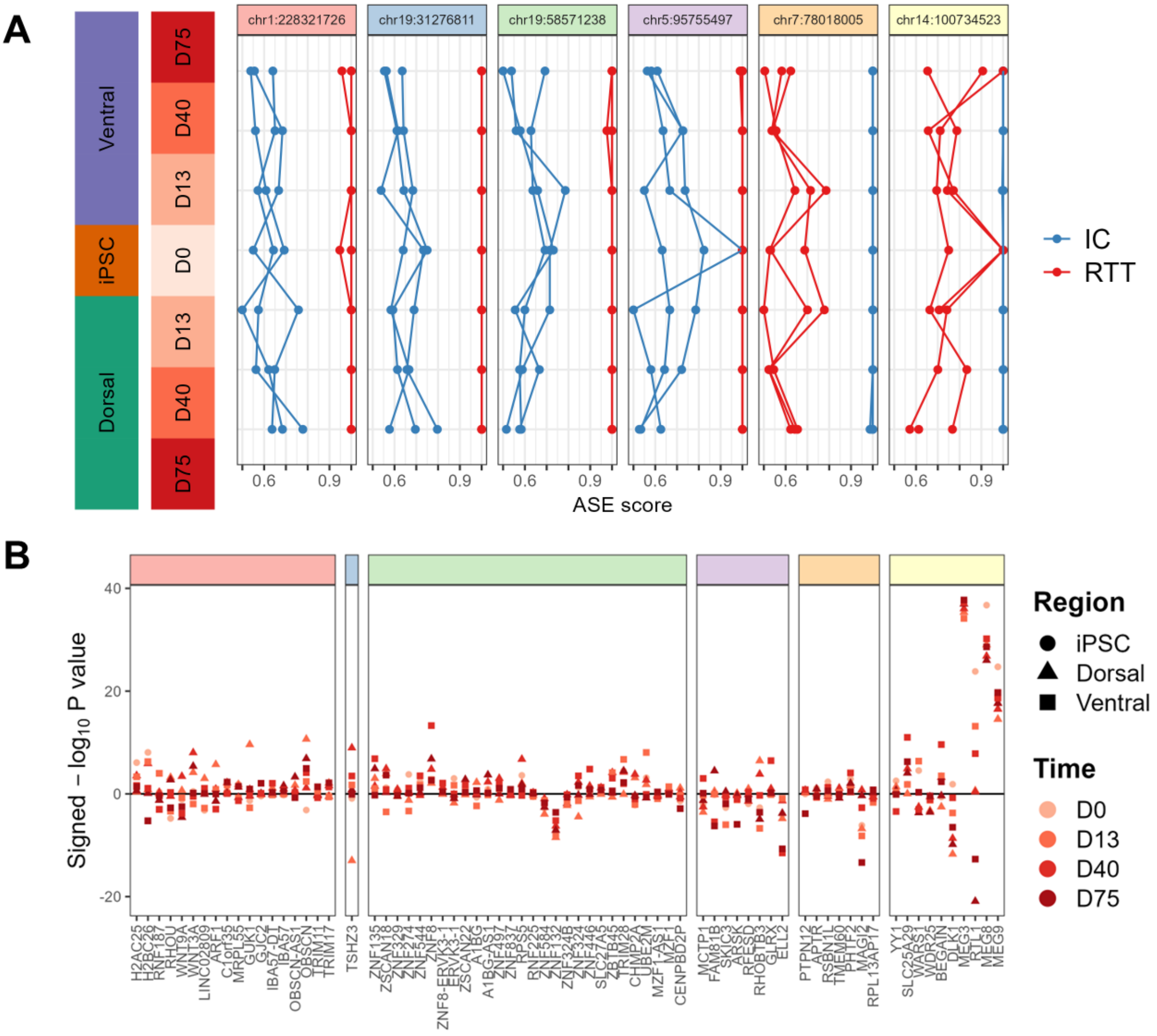

Figure S24. Results from the allele-specific expression (ASE) analysis.

(A) ASE score of six imprinted genes that demonstrate the loss or gain of bi-allelic expression in the RTT brain organoids. The ASE score is the ratio of the expression most abundant allele and the total expression of both alleles. An ASE score of 0.5 indicates bi-allelic expression, whereas a score of 1 indicates mono-allelic expression.

(B) The signed -$\log_{10}$ P value of the DEG analysis per time point and brain region is shown for the genes located within 500 kb from the SNPs of panel A. In this figure, the genes are sorted based on the genomic location of its transcription start site.

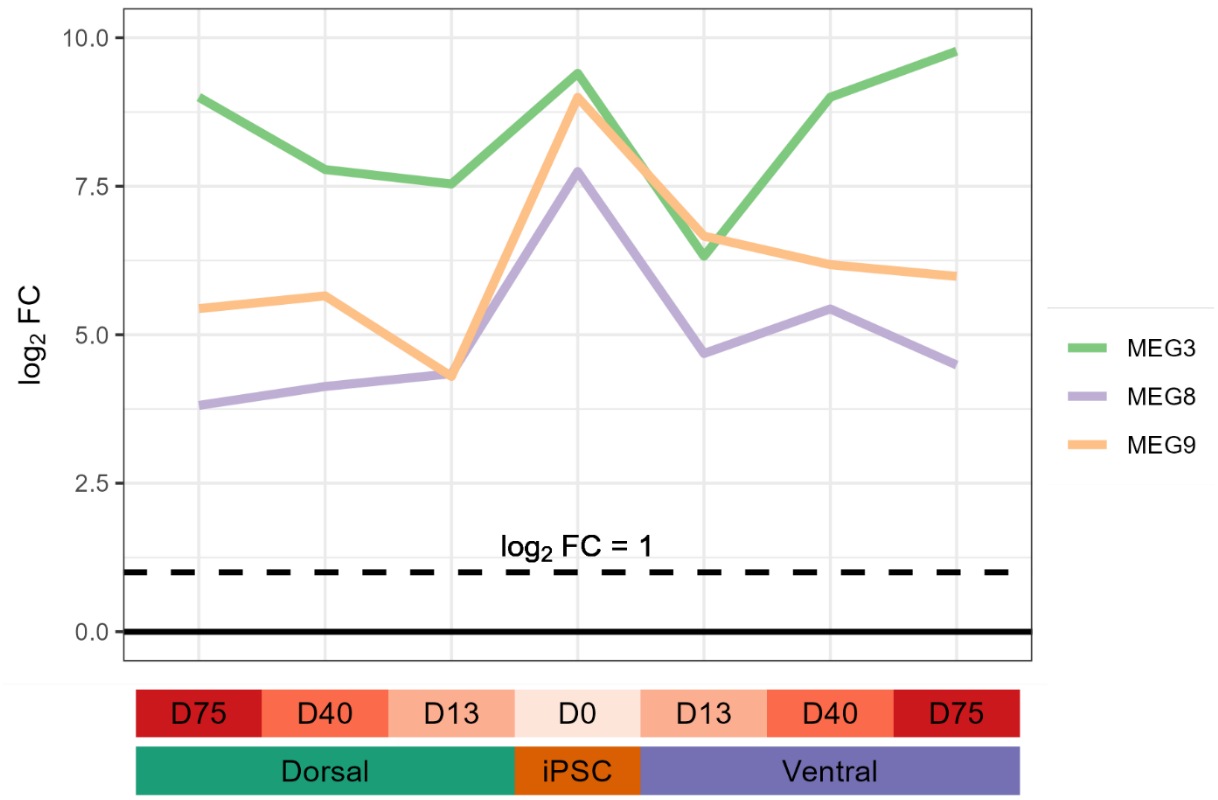

Figure S25. $\log_2$FCs per time point and brain region for the three genes from the imprinted *MEG* family. The $\log_2$FCs are consistently higher than 1, indicating that the increase cannot be solely attributed to the gain of bi-allelic expression.

Table S1. Selection criteria for the identification of time point- and region-specific RTT protein markers.

	Dorsal			iPSC	Ventral		
	D75	D40	D13	D0	D13	D40	D75
Dorsal region *	x	x	x	x			
Ventral region *				x	x	x	x
Early-time *			x	x	x		
Mid-time *		x	x		x	x	
Late-time *	x	x				x	x
Overall †	x	x	x	x	x	x	x

* Differentially expressed (*i.e.,* FDR-adjusted P value < 0.05 AND $|log_2FC| > 1$) with a consistent direction of effect in at least three of the time points and/or brain regions. † Differentially expressed (*i.e.,* FDR-adjusted P value < 0.05 AND $|log_2FC| > 1$) with a consistent direction of effect in at least six of the seven time points and/or brain regions.

Table S2. Imprinted genes with a bi-allelic expression status in the IC brain organoids. The strand of the reads is indicated by (+) for the plus strand and (-) for the minus strand.

Heterozygous SNP	Gene
chr1:228321726 (+)	*OBSCN*
chr5:95755497 (+)	*RHOBTB3*
chr7:50463289 (-)	*DDC*
chr7:94911104 (+)	*PPP1R9A*
chr7:151064227 (+)	*SLC4A2*
chr11:110165640 (+)	*ZC3H12C*
chr14:64170429 (+)	*SYNE2*
chr16:51138766 (-)	*SALL1*
chr16:66566576 (+)	*CMTM1*
chr19:31276811 (-)	*TSHZ3*
chr19:58571238 (-)	*MZF1*

Supplementary References

1. Gomes, A.R., Fernandes, T.G., Vaz, S.H., Silva, T.P., Bekman, E.P., Xapelli, S., Duarte, S., Ghazvini, M., Gribnau, J., Muotri, A.R., et al. (2020). Modeling Rett Syndrome With Human Patient-Specific Forebrain Organoids. Front Cell Dev Biol *8*, 610427. 10.3389/fcell.2020.610427.
2. Bahram Sangani, N., Koetsier, J., Gomes, A.R., Diogo, M.M., Fernandes, T.G., Bouwman, F.G., Mariman, E.C.M., Ghazvini, M., Gribnau, J., Curfs, L.M.G., et al. (2024). Involvement of extracellular vesicle microRNA clusters in developing healthy and Rett syndrome brain organoids. Cell Mol Life Sci *81*, 410. 10.1007/s00018-024-05409-7.
3. Bond, A.M., Vangompel, M.J., Sametsky, E.A., Clark, M.F., Savage, J.C., Disterhoft, J.F., and Kohtz, J.D. (2009). Balanced gene regulation by an embryonic brain ncRNA is critical for adult hippocampal GABA circuitry. Nat Neurosci *12*, 1020-1027. 10.1038/nn.2371.
4. Lund, I.V., Hu, Y., Raol, Y.H., Benham, R.S., Faris, R., Russek, S.J., and Brooks-Kayal, A.R. (2008). BDNF selectively regulates GABAA receptor transcription by activation of the JAK/STAT pathway. Sci Signal *1*, ra9. 10.1126/scisignal.1162396.
